# SARS-CoV-2 inhibits EBV-dependent tumor proliferation through PLpro-mediated degradation of EBNA1

**DOI:** 10.1016/j.apsb.2026.07.042

**Published:** 2026-07-30

**Authors:** Liubing Du, Mengxuan Wang, Xue Liu, Ziliang Peng, Zhihui Zhang, Zhanjia Zhang, Zhijuan Liang, Qingrong Sun, Jiaxin Zhuang, Kongyang Ma, Yao-Qing Chen, Panpan Hou, Hua Zhang, Deyin Guo, Ji-An Pan, Xiaoxue Peng

**Affiliations:** a The Molecular Cancer Research Center, School of Medicine, Shenzhen Campus of Sun Yat-sen University, Shenzhen 518107, China; b The Division of Basic Research, Guangzhou National Laboratory, Bio-island, Guangzhou 510320, China; c The Center for Infection and Immunity Study, School of Medicine, Shenzhen Campus of Sun Yat-sen University, Shenzhen 518107, China; d Shenzhen Key Laboratory for Systems Medicine in Inflammatory Diseases, School of Medicine, Shenzhen Campus of Sun Yat-sen University, Shenzhen 518107, China; e State Key Laboratory of Respiratory Disease, National Clinical Research Center for Respiratory Disease, Guangzhou Institute of Respiratory Health, The First Affiliated Hospital of Guangzhou Medical University, Guangzhou 510120, China; f School of Public Health (Shenzhen), Shenzhen Campus of Sun Yat-sen University, Shenzhen 518107, China

**Keywords:** SARS-CoV-2, EBV, PLpro, EBNA1, Viral co-circulation, Anti-EBV tumor therapy, EBV reactivation, COVID-19, Protein degradation, Protease activity assay

## Abstract

Co-circulation of multiple viruses within a host can produce pathological outcomes distinct from those of single-virus infections. Over 700 million documented severe acute respiratory syndrome coronavirus 2 (SARS-CoV-2) infections during the coronavirus disease 2019 (COVID-19) pandemic indicate frequent co-circulation of SARS-CoV-2 with other human viruses. However, how SARS-CoV-2 infection influences other viral infections remains unclear. In this study, we investigated how the proteolytic activity of SARS-CoV-2 papain-like protease (PLpro) influences infection by other human viruses. *In silico* analysis identified candidate PLpro cleavage sites across numerous viral proteins, and biochemical assays confirmed that PLpro catalyzes the degradation of Epstein–Barr virus (EBV) nuclear antigen 1 (EBNA1) in a manner requiring its protease activity. Both ectopic PLpro expression and SARS-CoV-2 infection reduced EBNA1 abundance, leading to reactivation of latent EBV. Given the essential role of EBNA1 in maintaining EBV-associated tumorigenesis, SARS-CoV-2 and PLpro-mediated degradation of EBNA1 can be translated into an inhibitory effect on EBV-driven tumor proliferation. These findings establish a molecular mechanism by which SARS-CoV-2 PLpro disrupts EBV latency and suppresses EBV-associated tumorigenesis, positioning EBNA1 as a potential therapeutic target in EBV-related malignancies.

## Graphical abstract

SARS-CoV-2 papain-like protease induces reactivation of latent Epstein-Barr virus and inhibits EBV-dependent tumor cell proliferation by promoting EBNA1 degradation.

**Figure:**
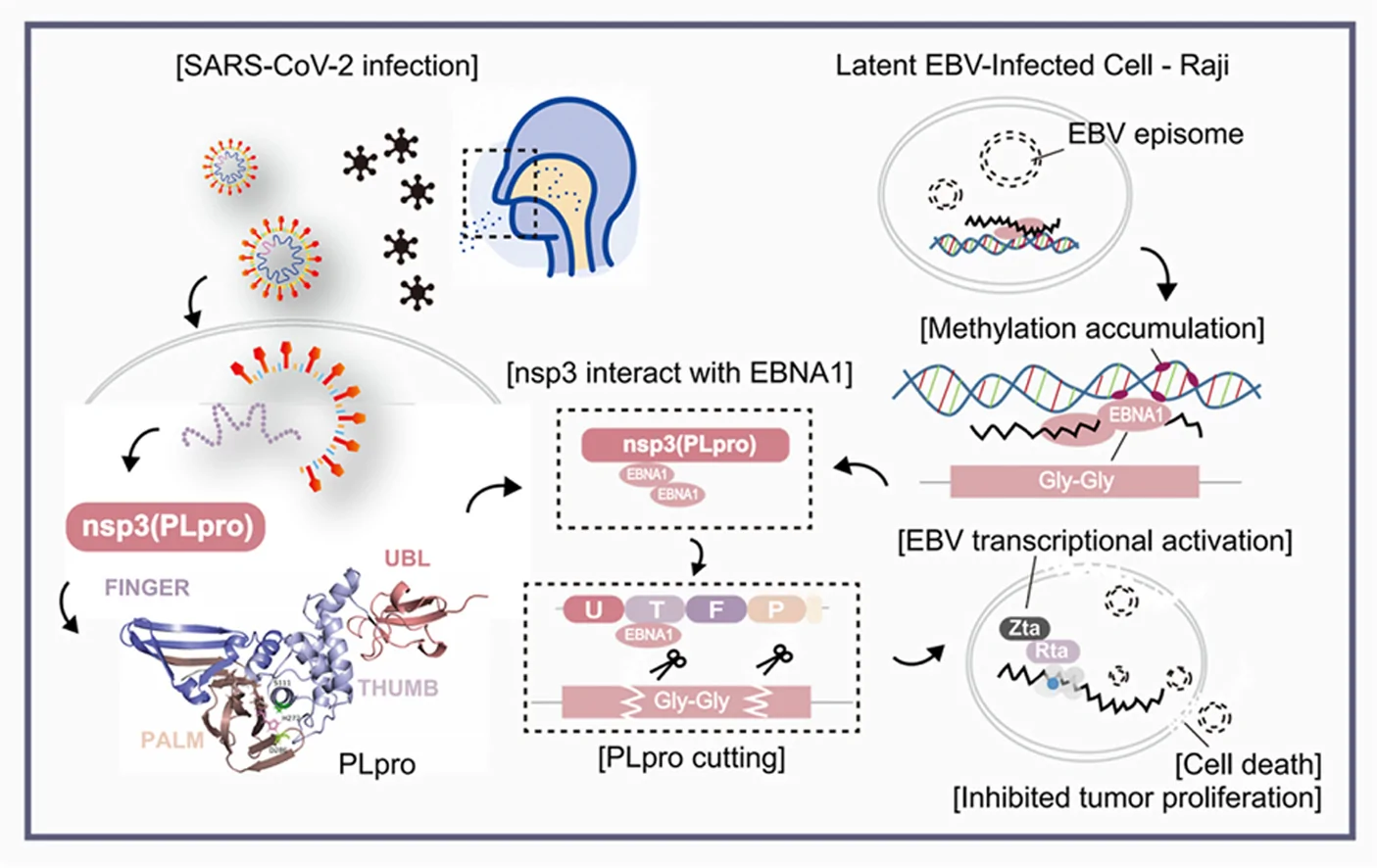

## 1 Introduction

The co-circulation of multiple viruses within a population can lead to simultaneous infections in a host, a phenomenon known as coinfection^1^, ^2^, ^3^. Coinfection can result in diverse patterns of virus–virus interaction, including interference, noninterference, synergy, and dependence assistance^4^. As one of the most devastating pandemics of this century, coronavirus disease 2019 (COVID-19) garnered significant attention from researchers. Numerous studies have been conducted across various aspects of COVID-19, including virology, epidemiology, diagnosis, and pathogenesis. However, research on the impact of COVID-19 on other viruses remains limited.

Severe acute respiratory syndrome coronavirus 2 (SARS-CoV-2), the causative pathogen of COVID-19, possesses the largest known viral RNA genome, encoding two long polypeptides^5^. These polypeptides are processed into 16 non-structural proteins (nsps) by their papain-like protease (PLpro) in nsp3^6^^,^^7^ and 3C-like protease (3CLpro) in nsp5^8^^,^^9^. Nsp7, nsp8, and nsp12 are involved in the assembly of the replication and transcription complex (RTC), which synthesizes genomic and subgenomic RNAs^10^. The viral subgenomic RNAs encode at least 10 viral accessory proteins and four structural proteins, including spike (S), envelope (E), membrane (M), and nucleocapsid (N)^11^.

Given their pivotal roles in the early event of viral replication, PLpro and 3CLpro were recognized as valuable targets for anti-viral drug development^12^^,^^13^. Besides that, its potential substrates have also been screened to enhance the understanding of the roles of these proteases in various biological processes^12^^,^^13^. However, it remains unknown whether these proteases can cleave proteins of other human viruses that co-infect with SARS-CoV-2. Should such cleavage occur, it could impair the replication of these viruses and thereby critically shape viral co-infection dynamics and co-circulation patterns.

Epstein–Barr virus (EBV) is a member of the *Gammaherpesvirinae* subfamily of the *Herpesviridae* family, thus also known as human herpesvirus 4 (HHV4), and latently infects nearly 90% of adults worldwide^14^. Saliva exchange contributes to the transfer of EBV among humans. Upon entering the host, EBV can infect epithelial cells of the oropharynx and stomach, as well as B cells through an unknown route using various receptors, such as Ephrin receptor A2 for epithelia^15^^,^^16^ and complement receptors 1 and 2 for B cells^17^^,^^18^. In the cell culture dish, EBV can transform human B cells into lymphoblastoid cell lines (LCLs), highlighting its tumorigenic potential^19^^,^^20^. Corresponding to its primary tropism for the cell types to infect, EBV infection can cause nasopharyngeal carcinoma, gastric carcinoma, and diffuse large B-cell lymphoma (DLBCL)^17^.

While EBV infection efficiently induces the transformation of B cells *in vitro*, EBV-associated epithelial tumors arise from the establishment of viral latent infection and the accumulation of premalignant genetic changes within cells^20^. EBV possesses a set of latent genes encoding several latent proteins and latent non-coding RNAs to facilitate its maintenance of latent infection, which is categorized as latency 0, latency I, latency II, and latency III^14^. As methylation changes in the viral genome, the viral lytic infection can be induced from latency 0 and latency I with the expression of viral genes associated with lytic replication. Among viral latent proteins, Epstein–Barr nuclear antigen 1 (EBNA1) is the only nuclear EBV protein expressed in both latent and lytic infections^21^, ^22^, ^23^. EBNA1 plays an essential role in suppressing spontaneous reactivation and lytic infections, highlighting an essential role of EBNA1 in EBV-associated tumorigenesis^21^.

In this study, we screened the proteomes of diverse human viruses for potential PLpro cleavage sites and uncovered a striking enrichment within EBNA1. Functional analyses revealed that SARS-CoV-2 infection triggers EBV reactivation by catalyzing the PLpro-mediated degradation of EBNA1. SARS-CoV-2 PLpro can recognize EBNA1 in the nucleus and perform cleavage on it. This PLpro-induced degradation of EBNA1 leads to a decrease in EBV-associated tumor proliferation. These findings highlight the potential role of SARS-CoV-2 PLpro in modulating EBV activity and suggest that EBNA1 may be a potential target in the interaction between these viruses.

## 2 Materials and methods

### 2.1 Cell cultures

HEK293T, HeLa, and Huh7.5 cells were cultured in DMEM (Cat# 11965, Gibco, Grand Island, New York, NY, USA), and Raji, BJAB, C666-1, and SNU-719 cells were cultured in RPMI-1640 (Cat# 11875093, Gibco). All media were supplemented with 10% fetal bovine serum (Cat A5670501, Gibco) and 1% penicillin/streptomycin (Cat# 15140122, Gibco). All cells were maintained at 37 °C in a 5% CO_2_ incubator and passaged using 0.25% trypsin-EDTA (Cat# 25200072, Gibco).

### 2.2 Plasmid construction and transfection

All oligos used in this study are listed in Supporting Information Table S1. SARS-CoV-2 replicon was cloned as described previously. The oligonucleotides encoding the EBNA1 or IRF3 short hairpin RNA were inserted into the pLKO.1 vector between the *Age*I and *Eco*RI sites. The EBNA1 sequence was synthesized (Sangon Biotech, Shanghai, China) and inserted into the LPC vector^49^. Nsp3 and PLpro (SARS-CoV-2, MN908947.3) were fused with GFP, Flag, or HA using MultiF Seamless Assembly Mix (Cat# RM20523, ABclonal, Wuhan, China) according to the manufacturer's instructions and inserted into the LPC vector. C111S, H272A, and D286S were generated with the site-directed mutagenesis strategy described previously^49^. The UB-Luci plasmid was constructed previously. The GFP-CAT sequence was obtained by fusing GFP and CAT genes with MultiF Seamless Assembly Mix and was cloned into the LPC vector.

For the plasmid transfection on HEK293T and HeLa cells, Hieff Trans® Liposomal Transfection Reagent (Cat# HB220930, Yeasen Biotechnology, Shanghai, China) was used following the manufacturer's instructions.

### 2.3 Virus genome collection and tetrapeptide motif analysis

Human pathogenic viruses were collected from the Human Virus Database (HVIDB, http://zzdlab.com/hvidb/) and screened according to the following criteria^50^: (1) confirmed human pathogenicity based on literature evidence; (2) availability of complete genome nucleotide and protein sequences in the NCBI GenBank database; and (3) complete coding sequence (CDS) annotations. Following screening, a total of 109 viruses were included for analysis.

Genome annotation information was retrieved from the GenBank database using the NCBI Entrez API *via* the Biopython library^51^, based on accession numbers. For each viral genome, all annotated CDS regions were extracted and translated into protein sequences using the standard genetic code. Quantitative analysis was performed on four tetrapeptide motifs: GG (two consecutive glycine residues), LXGG (leucine-any amino acid-two glycine residues), VXGG (valine-any amino acid-two glycine residues), and YXGG (tyrosine-any amino acid-two glycine residues), where X represents any of the 20 standard amino acids. The motif count for each virus represents the total number of identified tetrapeptide motifs across all CDSs in the genome. Subsequent statistical analysis and data visualization were conducted using the R packages tidyverse (v2.0.0) and ComplexHeatmap (version 2.20.0)^52^^,^^53^.

### 2.4 VRP packaging and infection

For the VRP packaging, Huh7.5 cells (5 × 10^6^) were transfected with 2 μg of VSV-G expressing plasmid^54^ and 10 μg of SARS-CoV-2 replicon^55^ using polyethyleneimine (PEI) reagent (DNA/PEI ratio, 1:4). Six hours post-transfection, the transfection mixture was replaced with fresh culture medium. The culture medium was harvested at 48, 72, and 96 h, clarified with centrifugation, aliquoted, and stored at −80 °C.

For VRP infection, the target cells were cultured in the culture medium containing VRPs and 8 μg/mL polybrene (Cat# H9268, Sigma–Aldrich, St. Louis, MO, USA). After 6 h of incubation, the culture medium was replaced with fresh medium. 24 or 48 h after infection, the cells were harvested. To examine the viral replication, the cell lysate was subjected to Western blot with anti-N antibody, or the total RNA was subjected to quantitative real-time PCR to detect viral genomic RNAs with the primers targeting viral leader and 5′ UTR sequence (leader) and viral subgenomic RNAs with the primers targeting viral leader and indicated gene sequence (M or N subgenomic RNA)^55^.

### 2.5 Lentiviral packaging and infection

For the lentiviral packaging^49^, HEK293T cells were transfected with psPAX2 (Cat# 12260, Addgene, Cambridge, MA, USA), pVSV-G, and pLKO.1-shRNAs or pLenti-Flag-GOI (gene of interest) at the ratio of 3:1:5 (μg) using Liposomal Transfection Reagent (Cat# HB220930, Yeasen Biotechnology, Shanghai, China). 48 h post-transfection, the culture medium was collected, filtered through 0.45 μm, aliquoted, and stored at −80 °C.

For lentiviral infection, the lentiviral stock was added to the culture medium containing 10 μg/mL polybrene. Six hours post-infection, the supernatant was replaced with fresh medium. After another 48 h of culture, the cell culture medium was replaced with the fresh medium containing 1 μg/mL puromycin. The puromycin-resistant cells were stored in liquid nitrogen.

### 2.6 Retroviral packaging and infection

For the retroviral packaging^49^, HEK293T cells were transfected with LPC-GOI (gene of interest), helper virus, and pVSV-G at the ratio of 2:1:0.8 (μg) using Liposomal Transfection Reagent (Cat# HB220930, Yeasen Biotechnology, Shanghai, China). 48 h post-transfection, the culture medium was collected, filtered through 0.45 μm, aliquoted, and stored at −80 °C.

For retroviral infection, retroviral stock was added to the culture medium containing 5 μg/mL polybrene. Six hours post-infection, the supernatant was replaced with fresh medium. After another 48 h of culture, the cell culture medium was replaced with the fresh medium containing 1 μg/mL puromycin. The puromycin-resistant cells were stored in liquid nitrogen.

### 2.7 AAV9 packaging

AAV9 virus was provided by OBiO Technology (Shanghai, China). Briefly, the PLpro sequence was inserted into pcAAV-CMV-MCS-WPRE, an AAV expression vector, to obtain the pAAV-PLpro plasmid, whose sequence was verified with DNA Sanger sequencing. pAAV-PLpro, pAAV-RC, and pHelper plasmids were transfected into AAV-293 cells at the ratio of 1:1:1 with a lipofectamine reagent. 72 h post-transfection, the cells were collected and frozen–thawed multiple times. The virus in the supernatant was purified and concentrated with ultracentrifugation and gradient ultracentrifugation. The virus titer was quantified with quantitative real-time PCR.

### 2.8 RNA isolation

Total RNA was extracted from cells or animal tissues, which were cut into small pieces using the RNA-easy Isolation Reagent (Cat# R701-02-AA, Vazyme, Nanjing, China) following the manufacturer's instructions. RNA concentration and purity were measured using a NanoDrop spectrophotometer (ThermoFisher Scientific, Waltham, MA, USA). RNAs were reverse-transcribed into cDNA using HiScript IV RT SuperMix for qPCR (Cat# R423-01, Vazyme).

### 2.9 Genomic DNA extraction

Total DNA was extracted using the PureLink™ Genomic DNA Kit (Cat# K182002, ThermoFisher Scientific) following the manufacturer's instructions. DNA concentration and purity were measured using a NanoDrop spectrophotometer (ThermoFisher Scientific). EBV DNA was quantified by qPCR using Taq Pro Universal SYBR qPCR Master Mix (Cat# Q712-02-AA, Vazyme).

### 2.10 Quantitative real-time PCR

Quantitative real-time PCR (qRT-PCR) was conducted using the Taq Pro Universal SYBR qPCR Master Mix (Cat# Q712-02, Vazyme) on a StepOnePlus Real-Time PCR System (Applied Biosystems, Carlsbad, CA, USA). The relative expression levels of target genes were analyzed using the 2^–ΔΔCt^ method, with GAPDH employed as the internal control.

### 2.11 Immunofluorescence (IF)

HeLa cells were cultured on glass coverslips in 24-well plates. After cell monolayers were formed and confluency reached 50%, the cells were subjected to transfection with the indicated plasmids. 48 h post-transfection, the cells were washed with PBS and fixed with 4% paraformaldehyde, which was pre-warmed at room temperature for 15 min. Next, the cells were washed twice with PBS and permeabilized in PBS containing 0.1% Triton X-100 for 10 min at room temperature. Next, the cells were incubated in a blocking buffer (PBS containing 0.05% Tween-20 and 5% goat serum) for 1 h at room temperature. Next, the cells were incubated with a blocking buffer containing primary antibodies overnight at 4 °C. On the second day, cells were washed with PBST (PBS containing 0.05% Tween-20) three times and incubated with a blocking buffer containing an anti-mouse secondary antibody (Cat# A-11005, Invitrogen, Carlsbad, CA, USA) for 1 h at room temperature. After the incubation, the cells were washed with PBST three times. Then, the cells were incubated with PBS containing 10 μg/mL DAPI (Cat# C1005, Beyotime Biotechnology, Shanghai, China) for 5 min in the dark. After being washed twice with PBS, the coverslips were mounted on glass slides. The cells were observed under the microscope (Zeiss Axio Observer 7, Oberkochen, Germany & EVOS M5000, ThermoFisher Scientific) or confocal microscope (Zeiss LSM900, Oberkochen, Germany).

### 2.12 Dual-luciferase reporter assay

HEK293T cells were seeded in 48-well plates. After an overnight recovery, the cells were transfected with pRL-TK, UB-Luci, and the indicated plasmids using Liposomal Transfection Reagent (Cat# HB220930, Yeasen Biotechnology, Shanghai, China). 48 h post-transfection, the cells were washed briefly with PBS and lysed in Passive Lysis Buffer (Cat# E1941, Promega, Madison, Wisconsin, USA). The firefly and renilla luciferase activities were measured using Dual-Glo® Luciferase Assay System (Cat# E1960, Promega, Madison, WI, USA) following the manufacturer's instructions. 20 μL of cell lysates were mixed with 10 μL of the firefly luciferase substrate and the renilla luciferase substrate sequentially. The firefly and renilla luciferase activities were measured on the plate reader (BioTek Synergy H1, Winooski, VT, USA).

### 2.13 Co-immunoprecipitation

HEK293T cells were transfected with indicated plasmids. 48–72 h post-transfection, cells were harvested and lysed in DISC IP lysis buffer (30 mmol/L Tris, pH 7.5, 150 mmol/L NaCl, 10% glycerol, 1% Triton X-100, 0.5 mmol/L EDTA, 10 mmol/L NaF, 100 μmol/L sodium orthovanadate, and 200 μmol/L PMSF) supplemented with cOmplete™, EDTA-free Protease Inhibitor Cocktail (Cat# 04693132001, Roche, Basel, Switzerland) on ice for 1 h. Cell lysates were cleared by centrifugation at 12,000×*g* (CF1542R, SCILOGEX, Rocky Hill, CT, USA) for 10 min at 4 °C. Protein concentrations were determined with a BCA protein assay (Cat# 23225, ThermoFisher Scientific). Cell lysates were added to 15 μL of anti-Flag Nanobody Magarose Beads (Cat# KTSM1338, AlpaLifeBio, Shenzhen, China) equilibrated with DISC IP lysis buffer. The mixtures were incubated overnight at 4 °C with gentle rotation. On the second day, the beads were washed five times with DISC IP lysis buffer. The proteins on beads were eluted by boiling in 2 × SDS loading buffer at 95 °C for 10 min.

### 2.14 Cellular fractionation

The cellular fractionation assay was conducted using a Nuclear and Cytoplasmic Protein Extraction (Cat# P0027, Beyotime Biotechnology, Shanghai, China) following the manufacturer's instructions. Briefly, cells were washed with ice-cold PBS, harvested by scraping, and pelleted at 800×*g* (CF1542R, SCILOGEX, Rocky Hill) for 5 min at 4 °C. The cell pellets were resuspended in buffer A containing 1 mmol/L PMSF (phenylmethylsulfonyl fluoride). After incubation on ice for 15 min, one-tenth volume of buffer B was added to the lysates. The lysates were mixed with 5 s of vortex and spun at 16,000×*g* (CF1542R, SCILOGEX, Rocky Hill) at 4 °C for 10 min. The supernatants, the cytoplasmic fraction, were recovered. The precipitates were resuspended in one-fourth volume of nuclear lysis buffer containing 1 mmol/L PMSF. The mixtures were incubated on ice at 4 °C for 30 min. During the incubation, the mixtures were mixed with 5 s of vortex every 5 min. Then, the mixtures were spun at 16,000×*g* (CF1542R, SCILOGEX, Rocky Hill) at 4 °C for 10 min. The supernatants, the nuclear fraction, were recovered.

### 2.15 Western blot

The cellular total proteins were extracted using RIPA buffer (Cat# CW2333S, CWBIO, Jiangsu, China) supplemented with 10 mmol/L NaF, 100 μmol/L sodium orthovanadate, and 200 μmol/L PMSF, and cOmplete™, EDTA-free Protease Inhibitor Cocktail. Protein concentrations were determined using a BCA protein assay (Cat# 23225, ThermoFisher Scientific). 30 μg of protein was separated by SDS-PAGE and transferred onto 0.45 or 0.22 μm nitrocellulose (NC) membranes (Cat# HATF00010, Sigma–Aldrich).

The membranes were blocked in PBS-T containing 5% nonfat milk (blocking buffer) for 1 h at room temperature and incubated in blocking buffer containing the primary antibodies overnight at 4 °C. After washing three times with PBS-T, membranes were incubated in blocking buffer containing fluorescent secondary antibodies for 1 h at room temperature. Then, the membranes were washed five times with PBS-T. Protein bands were visualized on a Li-COR Odyssey infrared imaging system (Li-COR Biosciences, Lincoln, NE, USA) or ChemiDoc MP Imaging System (Bio-Rad, Hercules, CA, USA).

The antibodies used in this study are listed below.

anti-SARS-CoV-2 Nucleocapsid (Npro) (Cat# 40143-R001, Sino Biological, Beijing, China)
anti-BZLF1 (Zta) (Cat# CSB-PA355980ZA01EFA, CUSABIO, Barksdale, DE, USA)
anti-EBV EBNA1 (1EB12) (Cat# sc-81581, Santa Cruz Biotechnology, Santa Cruz, CA, USA)
anti-GFP (Cat# ABT2021, Abbkine, Wuhan, China)
anti-6 × His (Cat# MA1-21315, ThermoFisher Scientific, Waltham, MA, USA)
anti-HA-mouse (Cat# AE008, ABclonal, Wuhan, China)
anti-HA-rabbit (Cat# 81290-1-RR, Proteintech, Wuhan, China)
anti-Flag (Cat# 66008-4-Ig, Proteintech, Wuhan, China)
anti-GAPDH (Cat# 10494-1-AP, Proteintech, Wuhan, China)
anti-*α*-Tubulin (Cat# 11224-1-AP, Proteintech, Wuhan, China)
anti-Histone H3 (Cat# sc-517576, Santa Cruz Biotechnology, Santa Cruz, CA, USA)
anti-Lamin B1 (Cat# AB16048, Cambridge, the UK)
anti-PARP (Cat# 9542, Cell Signaling Technology, Danvers, MA, USA)
anti-Caspase 7 (Cat# 67956-1-Ig, Proteintech, Wuhan, China)
anti-ACE2 (Cat#66699-1-Ig, Proteintech, Wuhan, China)
IRDye® 800CW Goat anti-Rabbit IgG Secondary Antibody (Cat# 926-32211, Li-COR Biosciences, Lincoln, NE, USA)
IRDye® 680CW Goat anti-Mouse IgG Secondary Antibody (Cat# 926-68070, Li-COR Biosciences)

### 2.16 Hematoxylin and eosin (H&E) staining

Mouse tumor tissue was fixed in 10% neutral formalin at room temperature for up to 24 h. The fixed tissue was then placed in an embedding box. The embedding box was immersed in distilled water for 1 h, 75% ethanol for 45 min, 85% ethanol for 45 min, 95% ethanol for 30 min, anhydrous ethanol for 20 min × 2, xylene for 30 min × 2, and 65 °C paraffin for 30 min × 2. Then, the tissue is embedded in paraffin. Paraffin tissue blocks were sectioned at 5 μm on a standard rotary microtome (Leica, Wetzlar, Germany). The sections were mounted on a glass slide and baked at 60–65 °C for 1–2 h.

The section slides were immersed in xylene I for 10 min, xylene II for 5 min, anhydrous ethanol for 5 min, 95% ethanol for 5 min, 85% ethanol for 5 min, 75% ethanol for 5 min, distilled water for 1 min, hematoxylin solution for 5 min, running water for 5 min, 0.1% hydrochloric acid and 95% ethanol for 30 s, running water for 1 min, the bluing solution for 1 min, running water for 5 min, 95% ethanol for 5–10 s, eosin for 1 min, running water for 3 min, 95% ethanol for 5 min, anhydrous ethanol for 3 min × 2, and xylene for 5 min × 2. The slides were sealed in a mounting medium.

### 2.17 Mice

Mice were housed in a pathogen-free facility. Raji cells were resuspended in RPMI-1640 medium, and cell concentrations were adjusted to 1 × 10^7^/100 μL. The cell suspension was mixed with Matrigel matrix (Cat# 40188ES08, Yeasen Biotechnology, Shanghai, China) at a ratio of 1:1. Subcutaneous injection of 1 × 10^7^ Raji cells was performed on six-week-old female mice at the inguinal regions. Five days post-subcutaneous injection, intratumoral injections were performed on the tumors with VRPs or AAV9. The body weight and the tumor volume were recorded. All animal experimental procedures were approved by the Institutional Animal Care and Use Committee (IACUC), Sun Yat-sen University (Approval No. SYSU-IACUC-2024-003087).

### 2.18 Statistical analysis

Western blot results were analyzed with Image Studio of Odyssey CLx. The amino acid consensus sequence was analyzed and presented with WebLogo. The immunofluorescence results were analyzed using NIS view and Image J. Statistical analyses were carried out using Microsoft Excel and GraphPad Prism (version 8). Data are expressed as mean ± standard error of the mean (SEM) from at least three independent experiments. Two-group comparisons were performed with an unpaired Student's *t*-test or one-way ANOVA with analysis. A *P*-value of less than 0.05 was considered statistically significant, and detailed *P*-values are provided in the figure legends. The figures were processed with PowerPoint 2016, Adobe Photoshop CC 2021, or Adobe Illustrator CC 2021.

## 3 Results

### 3.1 SARS-CoV-2 PLpro cleaves EBNA1 at multiple Gly–Gly-containing motifs

SARS-CoV-2 PLpro liberates nsp1, nsp2, and nsp3 from the viral polyprotein by cleaving at the nsp1/nsp2, nsp2/nsp3, and nsp3/nsp4 junctions, all of which contain the canonical LXGG↓XX motif (Fig. 1A). To test whether PLpro could similarly disrupt non-coronavirus human pathogens, we scanned the proteomes of a broad panel of human viruses for sequences matching the PLpro consensus (GG, LXGG, VXGG, and RXGG) (Fig. 1A and B). As expected, all experimentally confirmed coronavirus PLpro cleavage sites were accurately recovered. Notably, large DNA viruses-including human cytomegalovirus, human herpesvirus, molluscum contagiosum virus (MCV-1), Orf virus, vaccinia virus, and adenovirus-displayed marked enrichment of putative PLpro cleavage sites, likely due to their large genome size and coding regions. Among all the proteins identified bearing potential PLpro cleavage sites, EBV EBNA1 contained the highest density of predicted PLpro cleavage motifs (Fig. 1C and D).

**Figure 1:**
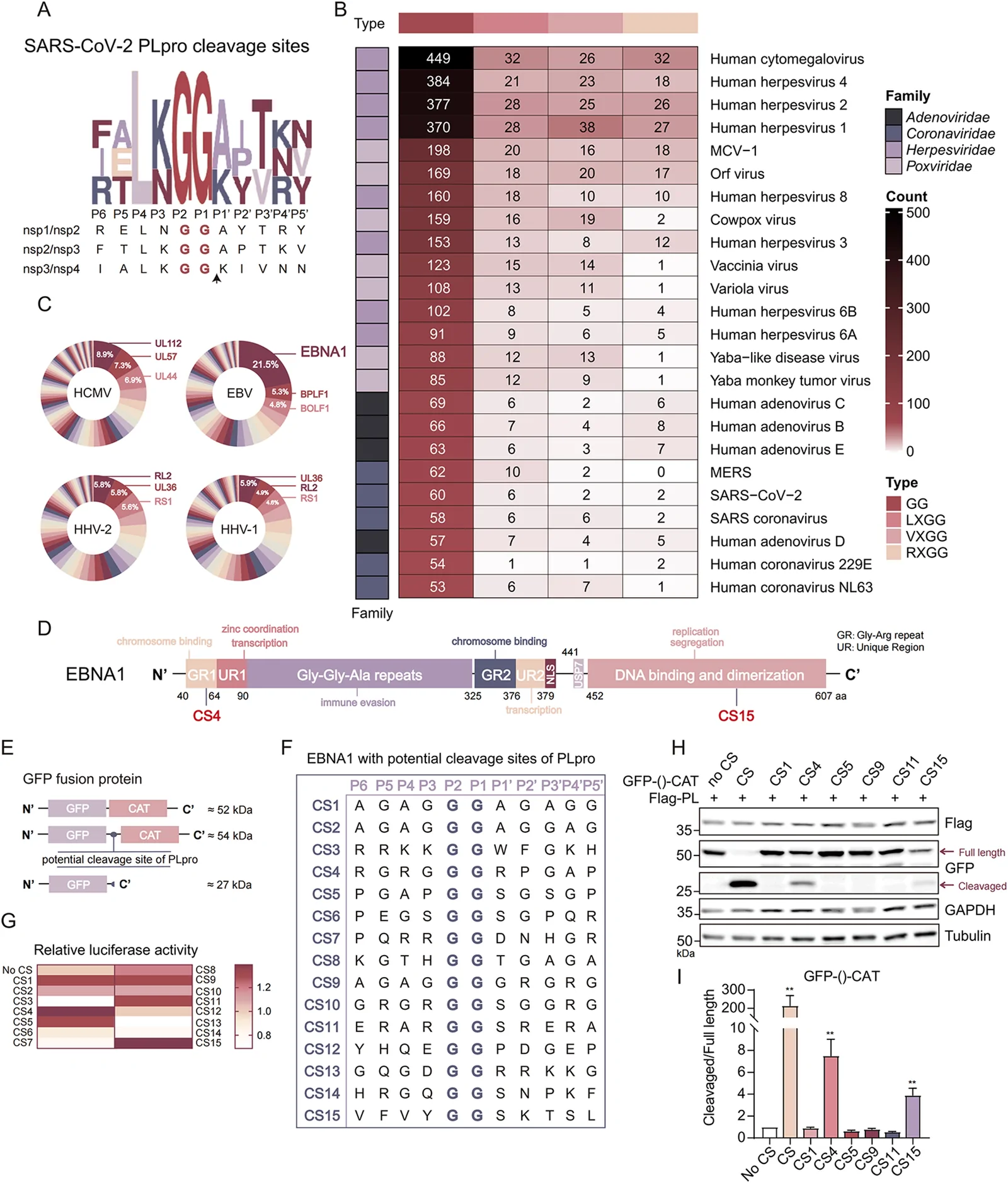
SARS-CoV-2 PLpro cleaves EBNA1 at multiple Gly–Gly-containing motifs. (A) Known papain-like protease (PLpro) cleavage sites in severe acute respiratory syndrome coronavirus 2 (SARS-CoV-2) were analyzed using WebLogo. (B) Four tetrapeptide motif types, GG, LXGG, VXGG, YXGG (where X denotes any amino acid), were used as bait to screen viral genomic sequences. The top heatmap represents the four PLpro cleavage motif types indicated in the figure. The left sidebar indicates viral family classifications. Numbers within cells represent the total counts of each motif type identified across all coding sequences within each viral genome. (C) The protein sequences of the top 4 viruses in (B) were analyzed; among them, the Epstein–Barr nuclear antigen 1 (EBNA1) protein of the Epstein–Barr virus (EBV) is the most GG motif-rich. (D) The schematic of the EBNA1 protein structure highlights the two potential PLpro cleavage sites (CSs), CS4 and CS15. (E) Schematic of the GFP-CAT-based PLpro activity reporter system. (F) Sequences of potential PLpro cleavage sites identified within EBNA1. (G) Cleavage efficiencies of 15 PLpro potential cleavage sites in EBNA1 were examined using the PLpro activity reporter system (*n* = 3). (H) HEK293T cells were co-transfected with the Flag-PL plasmid and various GFP-CS-CAT plasmids, containing the indicated CSs, and cell lysates were subjected to Western blot analysis using the indicated antibodies. Band intensities were quantified using Image Studio of Odyssey CLx, and the relative cleavage efficiency of each CS was obtained by calculating the ratio of GFP to GFP-CAT (*n* = 3) (I). The bars are represented as mean ± SEM. Statistics: unpaired two-tailed student's *t*-test or two-way ANOVA with multiple comparisons (∗∗*P* < 0.01).

To identify which sites are efficiently cleaved by PLpro, we developed a luciferase-based system and a GFP-CAT-based system (Fig. 1E and Supporting Information S1A). For the luciferase system, firefly luciferase (Luci) was fused to a polyubiquitin (UB) sequence that constitutively drives Luci to the proteasome for degradation. A candidate PLpro cleavage site (CS) was inserted in-frame between Luci and the ubiquitin sequence. When PLpro recognizes and cleaves the CS, Luci is liberated from the degradation signal; its luminescence therefore reports the efficiency of each candidate site (Fig. S1A). In the absence of PLpro, Luci is rapidly degraded; co-expression of active PLpro stabilizes Luci and boosts luminescence (Fig. S1B and S1C). PLpro-induced luciferase activity increase relied on its protease activity, as the mutation (C111S, H272A, or D286S) (Fig. S1D) or its protease inhibitor, GRL-0617 (Fig. S1E) (Gao, X. 2021), can suppress its function in promoting the luciferase activity. Employing this luciferase-based reporter system, we screened the top-ranked candidate cleavage sites (Fig. 1B) and found that most of the proteins contain at least one site efficiently processed by PLpro, including EBNA1 (Fig. S1F).

Because EBNA1 contains the greatest number of candidate PLpro sites (Fig. 1C and F), we concentrated on validating its cleavage. The luciferase reporter pinpointed CS4 and CS15 as the two sites most efficiently processed by PLpro among the 15 candidates (Fig. 1G). Although this system is ideal for high-throughput screening, the luminescence read-out is indirect and potentially confounded by variables unrelated to cleavage. To obtain direct evidence of site-specific proteolysis, we therefore established a Western-blot system in which each candidate site was inserted between GFP and chloramphenicol acetyltransferase (CAT) (Fig. 1E). PLpro-mediated cleavage was visualized as the appearance of the liberated GFP and CAT fragments (Fig. S1G). Processing required catalytically active PLpro, as the C111S, H272A, and D286S mutants impaired cleavage (Fig. S1H). Using this system, we re-evaluated the candidate sites prioritized by the luciferase screen and confirmed that CS4 and CS15 are cleaved by PLpro far more efficiently than the remaining sites (Fig. 1H and I).

### 3.2 SARS-CoV-2 PLpro activity drives the reduction of EBNA1 protein levels

Because PLpro is an enzymatic domain embedded within SARS-CoV-2 nsp3 (Fig. 2A), we first asked whether full-length nsp3 could cleave EBNA1. Expression of either nsp3 (Fig. 2B and C) or the isolated PLpro domain (Fig. 2D and E) markedly reduced EBNA1 levels, indicating that the PLpro domain alone recapitulates nsp3's capacity to cleave and degrade EBNA1. Owing to nsp3's large size (1945 aa) and low expression efficiency, subsequent experiments were performed with the standalone PLpro construct.

**Figure 2:**
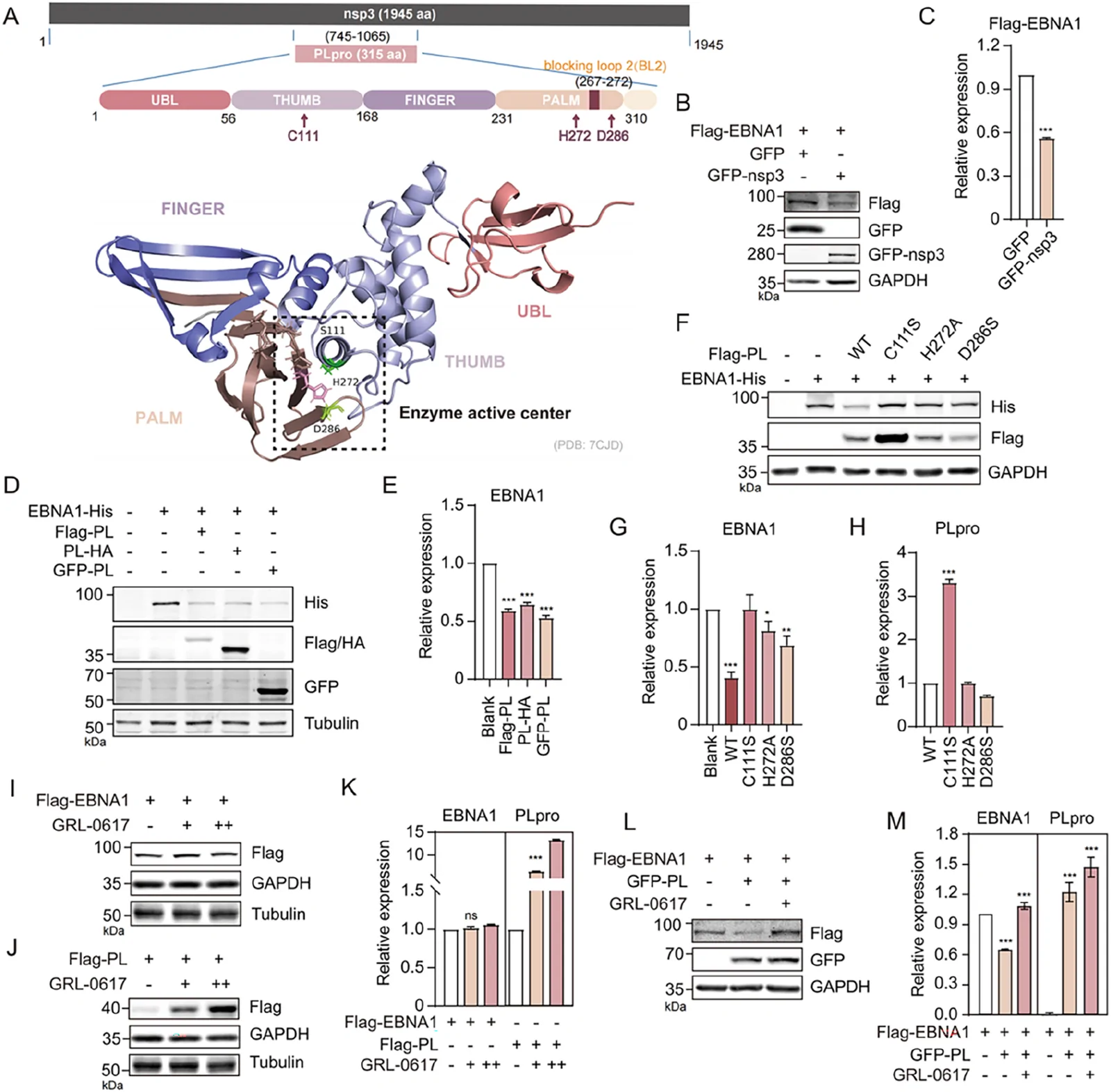
SARS-CoV-2 PLpro activity drives the reduction of EBNA1 protein levels. (A) The schematic of the nsp3 protein showing the location of PLpro and its four main domains, as well as key amino acid residues for its protease activity. The expressions of Flag-EBNA1 and GFP-nsp3 were analyzed by Western blot (B). The intensity of bands was quantified with Image Studio of Odyssey CLx (*n* = 3) (C). HEK293T cells were transfected with the EBNA1-His plasmid and either the Flag-PL, PL-HA, or GFP-PL plasmid. The expressions of EBNA1-His, Flag-PL, PL-HA, and GFP-PL were analyzed by Western blot (D). The intensity of bands was quantified using Image Studio of Odyssey CLx (*n* = 3) (E). HEK293T cells were transfected with the EBNA1-His plasmid and either the WT Flag-PL or Flag-PL with indicated mutations. The expressions of EBNA1-His and various Flag-PLs were analyzed by Western blot (F). The intensity of bands was quantified using Image Studio of Odyssey CLx (*n* = 3) (G, H). HEK293T cells were transfected with either the Flag-EBNA1 plasmid or Flag-PL, 24 h post-transfection, the cells were treated with GRL-0617 at 50 or 100 μmol/L for 48 h. Cells were lysed and subjected to Western blot analysis (I, J). The intensity of bands was analyzed and quantified using Image Studio of Odyssey CLx (*n* = 3) (K). HEK293T cells were transfected with the Flag-EBNA1 plasmid, GFP-PL plasmids, or the combination of the Flag-EBNA1 and GFP-PL plasmids. 24 h post-transfection, the cells were treated with GRL-0617 at 50 μmol/L for 48 h. The expressions of Flag-EBNA1 and GFP-PL were analyzed by Western blot (L). The intensity of bands was quantified using Image Studio of Odyssey CLx (*n* = 3) (M). Data are presented from at least three independent experiments with consistent results. The bars are represented as mean ± SEM. Statistics: unpaired two-tailed student's *t*-test or two-way ANOVA with multiple comparisons (∗*P* < 0.05, ∗∗*P* < 0.01, ∗∗∗*P* < 0.001).

We previously established that PLpro catalytic activity is indispensable for cleavage at the tested sites (Fig. S1D and S1H). We next examined whether PLpro enzymatic activity is strictly required for EBNA1 degradation. Our results showed that the mutation of C111S, H272A, or D286S can inhibit PLpro-mediated EBNA1 degradation (Fig. 2F–H and Supporting Information Fig. S2A). PLpro's protease inhibitor, GRL-0617, cannot alter the expression of EBNA1 (Fig. 2I and K). Despite increasing the protein level of PLpro (Fig. 2J and K), GRL-0617 can still inhibit PLpro-mediated EBNA1 degradation (Fig. 2L and M, Fig. S2B). To further confirm that degradation is cleavage site-dependent, we constructed EBNA1 mutants with mutations at the two identified key cleavage sites. We found that these mutations largely impaired PLpro-mediated EBNA1 degradation (Fig. S2C and S2D). In summary, our data demonstrate that PLpro activity is responsible for PLpro-mediated EBNA1 degradation.

### 3.3 SARS-CoV-2 PLpro localizes to the nucleus and physically interacts with EBNA1

EBNA1 is a nuclear protein expressed throughout both latent and lytic EBV infection (Fig. 3A)^21^, ^22^, ^23^. To investigate how nsp3 regulates EBV expression, we first explored whether nsp3 and EBNA1 share the same subcellular compartment. We ectopically expressed nsp3 or its PLpro domain in multiple cell lines and analyzed their localization by cellular fractionation (Fig. 3B and C) and immunofluorescence (Fig. 3D, Fig. S2E and S2F). The results showed that both nsp3 and PLpro were detected in the nucleus. Importantly, the expression of nsp3 and PLpro disrupted the EBNA1 aggregates, which are formed by the recruitment of EBNA1 at the chromosomal fragile site (Fig. 3D)^24^.

**Figure 3:**
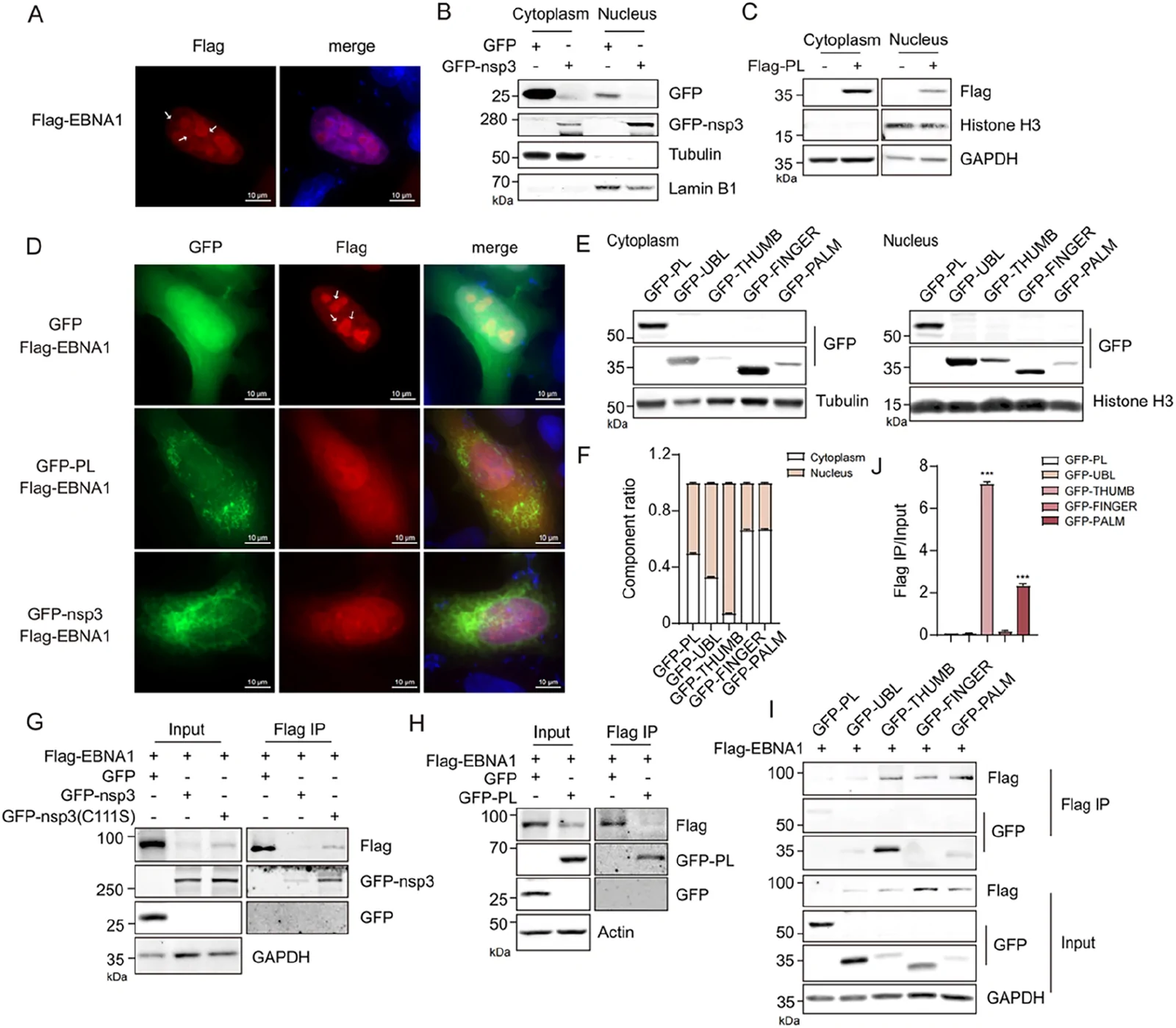
SARS-CoV-2 PLpro localizes to the nucleus and physically interacts with EBNA1. (A) HeLa cells were transfected with the Flag-EBNA1 plasmid and then fixed and stained with Flag antibody and DAPI. The cells were observed and photographed under a microscope. White arrows indicated the EBNA1 aggregates. HEK293T cells were transfected with GFP-nsp3 and Flag-PL. The cytoplasmic and nuclear fractions were analyzed by Western blot (B, C). (D) HeLa cells were transfected with Flag-EBNA1 and GFP, GFP-PL, or GFP-nsp3, followed by immunofluorescence analysis. HEK293T cells were transfected with the GFP-PL, GFP-UBL, GFP-THUMB, GFP-FINGER, or GFP-PALM plasmids. The cytoplasmic and nuclear fractions were analyzed by Western blot (E). The intensity of bands was quantified using Image Studio of Odyssey CLx, and the distribution of these five proteins in the cytoplasm and the nucleus was calculated (*n* = 3) (F). HEK293T cells were transfected with the Flag-EBNA1 plasmid and GFP, GFP-nsp3, or GFP-nsp3 (C111S) plasmid and subjected to immunoprecipitation (IP) assay with Flag agarose. The IP samples were analyzed using Western blot (G). HEK293T cells were transfected with the Flag-EBNA1 plasmid and either the GFP or GFP-PL plasmid and subjected to an immunoprecipitation (IP) assay with Flag agarose. The IP samples were analyzed using Western blot (H). HEK293T cells were transfected with the Flag-EBNA1 plasmid and either the GFP-PL, GFP-UBL, GFP-THUMB, GFP-FINGER, or GFP-PALM plasmid and subjected to an immunoprecipitation (IP) assay with Flag agarose. The IP samples were analyzed using Western blot (I). The intensity of bands was quantified using Image Studio of Odyssey CLx, and the relative pull-down efficiency of each sample was presented as the relative ratios of IP to input (*n* = 3) (J). The bar graphs showed the data of three independent experiments, presented as mean ± SEM. Statistics: unpaired two-tailed student's t-test or two-way ANOVA with multiple comparisons (∗∗∗*P* < 0.001).

The well-characterized PLpro structure comprises four principal domains, including the UBL, THUMB, FINGER, and PALM domains (Fig. 2A)^25^^,^^26^. To identify which domain(s) of PLpro govern nuclear targeting, we individually expressed each domain and mapped its subcellular distribution. The results showed that all four domains, like full-length PLpro, were detected in both cytoplasm and nucleus (Fig. 3E), yet their intracellular partitioning differed markedly (Fig. 3E and F). The UBL and THUMB domains accumulated preferentially in the nucleus, whereas the FINGER and PALM domains were predominantly cytoplasmic (Fig. 3E and F). Furthermore, we constructed GFP-fused PLpro deletion mutants lacking the UBL, THUMB, FINGER, or PALM domain, respectively. All PLpro deletion mutants showed nuclear localization (Fig. S2G and S2H). Notably, deletion of the FINGER domain resulted in the highest nuclear distribution (Fig. S2G and S2H), consistent with the data showing that the FINGER domain was least enriched in the nuclear fraction (Fig. 3E and F).

To establish a direct association between PLpro and EBNA1, we performed co-immunoprecipitation. Although the full-length nsp3–EBNA1 interaction was difficult to detect, the catalytically inactive nsp3 (C111S) mutant readily co-precipitated with EBNA1 (Fig. 3G), suggesting that catalytically active nsp3 may rapidly dissociate after cleaving EBNA1. Presumably because PLpro is expressed far more robustly than full-length nsp3, its interaction with EBNA1 is readily detectable (Fig. 3H). Mapping this interaction revealed that the THUMB domain of PLpro displayed the strongest affinity for EBNA1, whereas the other three domains showed weaker or negligible binding (Fig. 3I and J).

### 3.4 SARS-CoV-2 PLpro induces EBV reactivation in cells with latent EBV infection

Given the essential role of EBNA1 in both latent and lytic infections of EBV (Fig. 4A) and our finding that PLpro degrades EBNA1 (Fig. 2), we asked whether PLpro can disrupt EBV latency. We examined three established cell lines harboring latent EBV infection: SNU-719, a naturally derived gastric cancer cell line^27^; C666-1, a cell derived from a nasopharyngeal cancer (NPC) xenograft^28^; and Raji, a lymphoblast-like cell isolated from a Burkitt's lymphoma patient^29^. These three lines model the most prevalent EBV-driven malignancies. To further validate the susceptibility of these EBV latently infected cells to SARS-CoV-2 infection, we examined the expression of ACE2 protein in these cell lines (Fig. S2I). The results indicate that SARS-CoV-2 has the potential to infect host cells harboring latent EBV *in vivo*.

**Figure 4:**
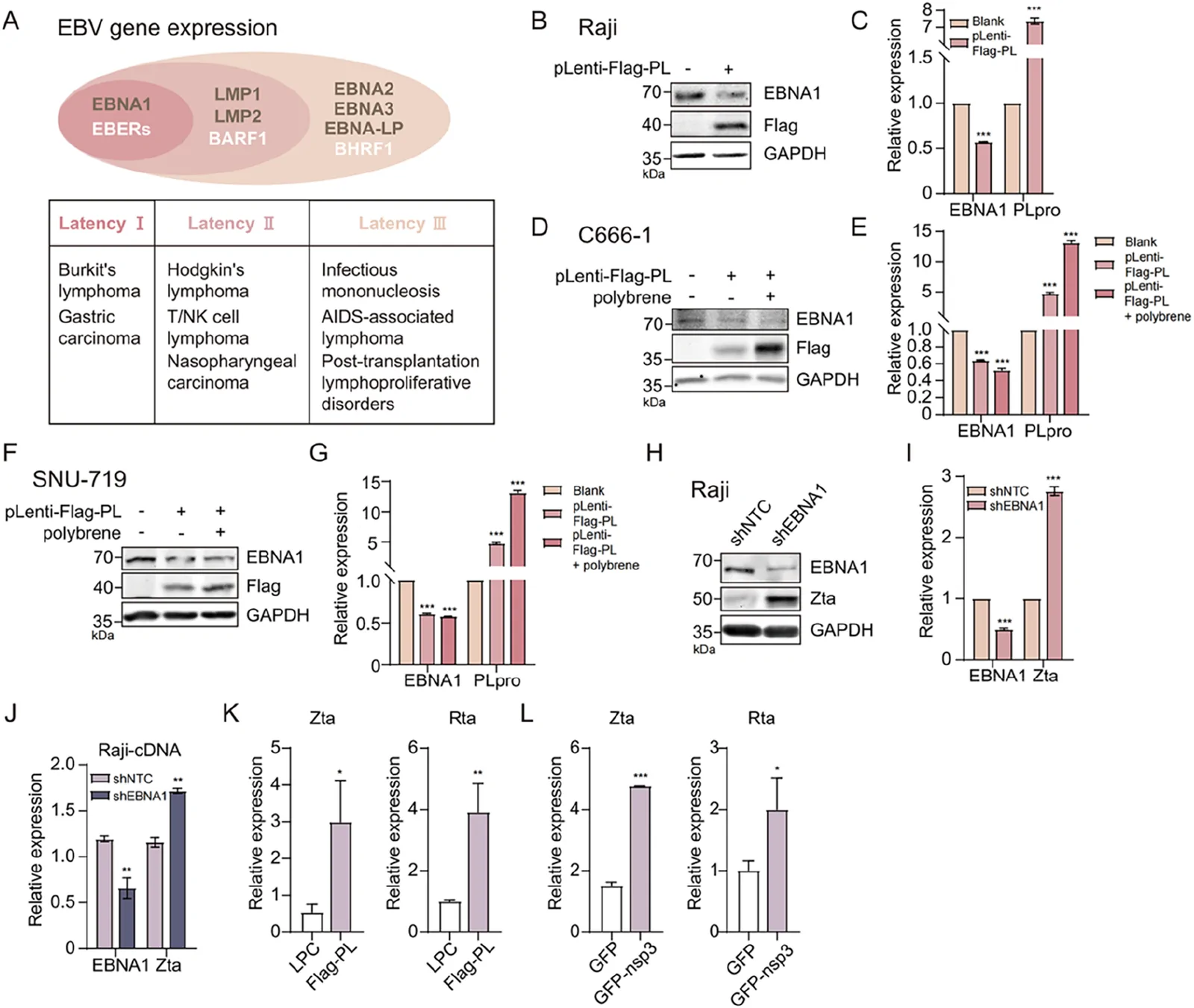
SARS-CoV-2 PLpro induces EBV reactivation in cells with latent EBV infection. (A) The schematic diagram of three types of EBV latency. Note that EBNA1 and Epstein-Barr virus—encoded small RNAs (EBERs) are expressed throughout the viral latent infection. Raji, C666-1, and SNU-719 cells were infected with lentivirus encoding Flag-PL. After infection, cells were treated with puromycin. Surviving cells were lysed in the DISC IP buffer. The expressions of EBNA1 and Flag-PLs were analyzed by Western blot (B, D, and F). The intensity of bands was quantified using Image Studio of Odyssey CLx (*n* = 3) (C, E, and G). Raji cells were infected with lentivirus encoding shEBNA1. Cell lysates and total RNA were analyzed by Western blot (*n* = 3) (H, I) and quantitative real-time PCR (*n* = 3) (J), respectively. Raji cells were infected with retroviruses encoding LPC or Flag-PL. Total RNA was extracted from cells and subjected to quantitative real-time PCR (*n* = 3) (K). Raji cells were infected with retroviruses encoding GFP or GFP-nsp3. Total RNA was extracted from stable cells and subjected to quantitative real-time PCR (*n* = 3) (L). Data are presented from at least three independent experiments with consistent results. The bars are represented as mean ± SEM. Statistics: unpaired two-tailed student's *t*-test or two-way ANOVA with multiple comparisons (∗*P* < 0.05, ∗∗*P* < 0.01, ∗∗∗*P* < 0.001).

We first assessed the effect of PLpro expression on the protein levels of EBNA1 in these three cell lines. We introduced the PLpro expression with the lentiviral system. As predicted, the PLpro expression decreased the protein levels of EBNA1 (Fig. 4B–G).

EBV latent infection is reactivated to lytic infection with the expression of the immediate-early transactivator Zta, which is also known as BZLF1, ZEBRA, Z, and EB1^21^^,^^30^. Zta preferentially binds the methylated promoter of the immediate-early gene Rta and activates its transcription^31^. Then, Zta and Rta act in concert to up-regulate a cohort of early lytic genes, driving the switch from EBV latency to full lytic replication^32^.

EBNA1 is essential for maintaining EBV latency and for suppressing spontaneous reactivation^21^. Consistent with the previous results, reducing the expression of EBNA1 using shRNA targeting EBNA1 (Fig. 4H–J) markedly induced the Zta expression^33^^,^^34^. We therefore tested whether PLpro, by degrading EBNA1, could similarly trigger reactivation. Indeed, compared to the vector control, expression of either PLpro (Fig. 4K) or full-length nsp3 (Fig. 4L) concomitantly elevated Zta and Rta levels, demonstrating that PLpro induces EBV reactivation in the cells with latent EBV infection.

### 3.5 SARS-CoV-2 infection induces EBV reactivation

Next, we asked whether SARS-CoV-2 infection, like ectopic PLpro or nsp3, can induce EBV reactivation in the cells with latent EBV infection.

To circumvent biosafety constraints while preserving authentic viral replication, we employed a single-cycle infectious viral replicon particle (VRP) system of SARS-CoV-2 to conduct assays in this study (Supporting Information Fig. S3A). The VRPs in this system were assembled in Huh7.5 cells, transfected with the replicon of SARS-CoV-2 lacking the S gene, constructed previously, as well as a VSV-G-expressing plasmid, and are released from the cells post-transfection. The structure of VRPs closely resembles that of authentic SARS-CoV-2, except that VRPs use VSV-G instead of S as the primary envelope protein for the viral entry. With VSV-G, VRPs could infect mammalian cells efficiently, and with SARS-CoV-2 genomic RNA in the viral particle, VRP infection results in the synthesis of all subgenomic RNAs except S subgenomic RNA, thereby encoding most viral proteins except the S protein. Thus, VRPs could behave similarly to authentic viruses within cells, and meanwhile, without the S gene in VRP genomic RNA, VRP infection cannot lead to the generation of progeny viruses.

First, we infected Raji cells with VRPs to confirm viral replication and assess effects on latent EBV. Our results showed that VRP infection results in the synthesis of viral genomic RNAs, as shown by the detection of 5′ UTR sequence (leader) (Fig. 5A), and subgenomic RNAs, as shown by the detection of M (Fig. S3B) and N (Fig. 5B) subgenomic RNAs, confirming that the VRP infection recapitulates authentic SARS-CoV-2 replication within infected cells. As with ectopic PLpro or nsp3, VRP infection strongly up-regulated Zta and Rta transcription, demonstrating that SARS-CoV-2 replication directly drives EBV reactivation in latently infected cells (Fig. 5C and Fig. S3B). Consistent with our results showing that PLpro induces the degradation of EBNA1 protein, VRP infection did not alter the mRNA level of EBNA1 (Fig. S3B).

**Figure 5:**
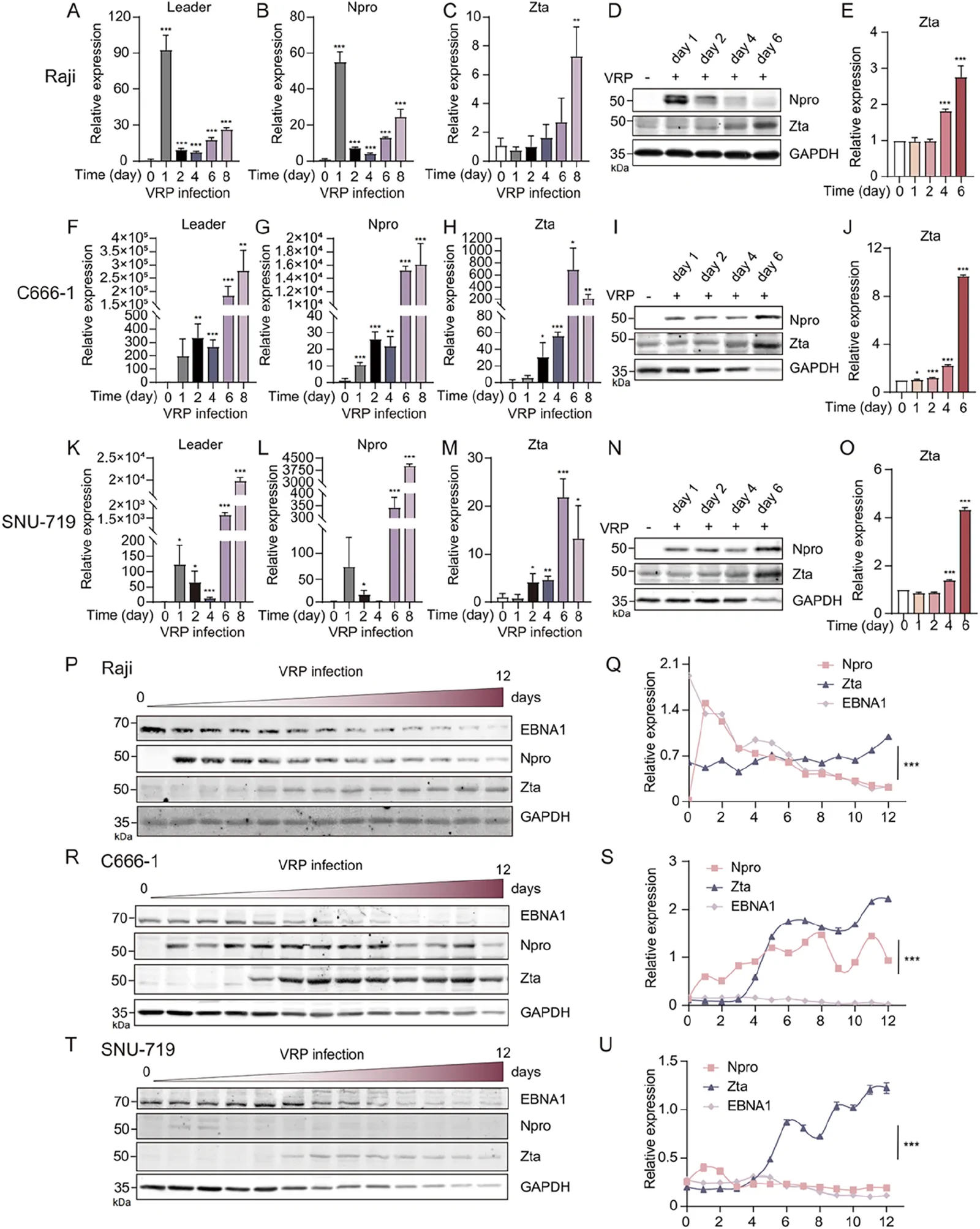
SARS-CoV-2 infection induces EBV reactivation. Raji, C666-1, and SNU-719 cells were infected with VRPs. On the indicated days post-infection, the cells were collected. The total RNA was extracted from one portion of the cells (*n* = 3). The viral genomic RNA (A, F, and K) and subgenomic RNA of Npro (B, G, L) and Zta mRNA (C, H, M) were measured using quantitative real-time PCR. The other portion of the cells was lysed in DISC IP buffer, and the cell lysates were analyzed by Western blot (D, I, and N). The band intensities were analyzed and quantified using Image Studio of Odyssey CLx (*n* = 3) (E, J, and O). One portion of the cells was lysed in DISC IP buffer, and the cell lysates were analyzed by Western blot (P, R, and T). Band intensities were analyzed and quantified using Image Studio of Odyssey CLx (*n* = 3) (Q, S, and U). Data are presented from at least three independent experiments with consistent results. The bars are represented as mean ± SEM. Statistics: unpaired two-tailed student's *t*-test or two-way ANOVA with multiple comparisons (∗*P* < 0.05, ∗∗*P* < 0.01, ∗∗∗*P* < 0.001).

We next examined how VRP infection affects Zta protein levels in Raji (Fig. 5D and E), C666-1 (Fig. 5I and J), and SNU-719 (Fig. 5N and O) cells. Robust replication of VRPs in all three cell lines was confirmed by detection of genomic (Leader) and sub-genomic (N) RNAs and of the N protein (Fig. 5A–O). VRP infection progressively reduced EBNA1 protein (Fig. 5P–U), and this decrease was mirrored by a concomitant rise in Zta, indicating that SARS-CoV-2-triggered EBV reactivation is contingent upon the loss of EBNA1.

To corroborate that SARS-CoV-2 infection drives EBV into lytic replication, we monitored gp350, the major envelope glycoprotein expressed during EBV reactivation. In contrast to Raji cells, which displayed only a modest upregulation of gp350 (Fig. S3B), C666-1 and SNU-719 cells infected with VRPs exhibited robust gp350 expression (Fig. S3C and S3D) along with a corresponding increase in EBV copy numbers (Fig. S3E and S3F), confirming that SARS-CoV-2 infection triggers EBV reactivation.

To further confirm that SARS-CoV-2 infection induces EBV lytic replication *via* EBNA1, we overexpressed EBNA1 in VRP-infected cells and found that EBNA1 expression attenuated VRP infection-induced Zta and Rta expression, indicating an indispensable role of EBNA1 in SARS-CoV-2 infection-induced EBV reactivation (Supporting Information Fig. S4A). In addition to PLpro, SARS-CoV-2 3CLpro also possesses protease activity; however, it is not involved in EBV reactivation, as 3CLpro expression failed to promote Zta and Rta expression to the same extent as PLpro (Fig. S4B). IRF3, a key transcription factor in host immune regulation, is well known to be cleaved by PLpro^35^. However, our results showed that knockdown of IRF3 did not alter SARS-CoV-2-induced EBV reactivation, further confirming the dominant role of the PLpro–EBNA1 axis in this reactivation (Fig. S4C).

### 3.6 SARS-CoV-2 inhibits EBV tumor proliferation

Having established that SARS-CoV-2 triggers EBV reactivation, we next asked whether this reactivation influences EBV-driven tumor growth. We first infected Raji cells with VRPs (Supporting Information Fig. S5A) and then delivered the Raji cells to BALB/c Nude mice with the groin injection (Fig. S5B). Twenty days post-injection, body weights were indistinguishable between groups (Fig. S5C), yet tumors arising from VRP-infected cells were markedly smaller (Fig. S5D–S5F). These data suggest that SARS-CoV-2 infection could influence the EBV tumor proliferation. However, closer inspection revealed that VRP infection altered Raji-cell morphology and impaired their proliferation *in vitro* (Fig. S5A, and Fig. 6A–C), accounting for the attenuated tumor growth rather than a direct oncolytic effect on established tumors.

**Figure 6:**
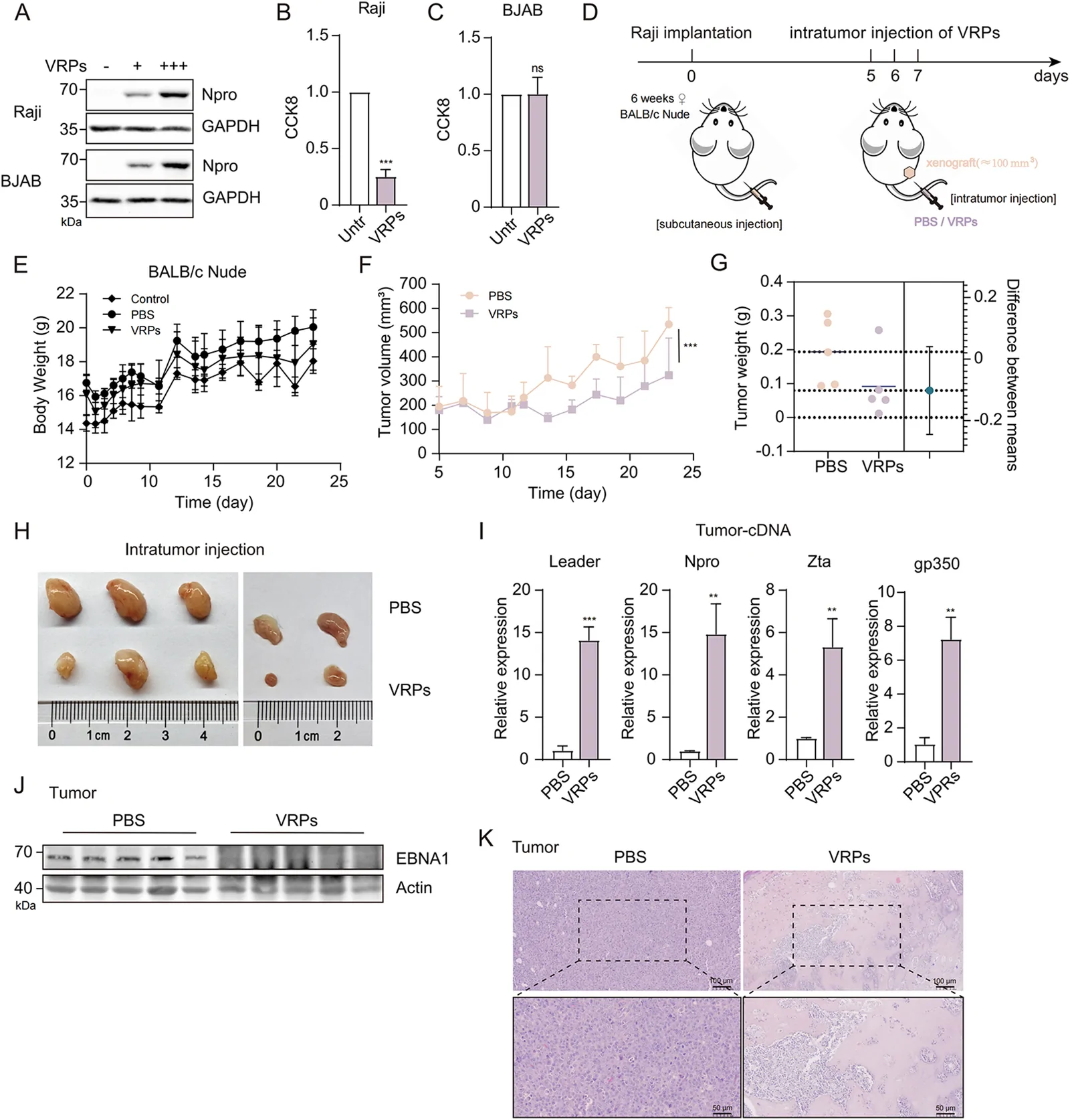
SARS-CoV-2 inhibits EBV tumor proliferation. BJAB and Raji cells were infected with low and high doses of VRPs. Two days post-infection, one portion of cells was harvested and lysed in RIPA buffer, and the cell lysates were subjected to Western blot analysis (A). Six days post-infection, the other portion was subjected to CCK-8 assay to assess cell viability (*n* = 3) (B, C). (D) The schematic illustration of the Raji xenograft mouse model with the infection of VRPs. Subcutaneous injections of 1 × 10^7^ Raji cells were performed on the 6-week-old female mice at the inguinal regions (*n* = 5). Five days post-subcutaneous injection, intratumoral injections were performed on the tumors in the right inguinal region with PBS or VRPs. The body weight (E) and the tumor volume (F) were recorded for 23 days. At the endpoint of the experiment, the tumors were collected, and tumor weight was recorded (G, H). (I) Total RNA in the tumor was extracted, reverse-transcribed, and analyzed by quantitative real-time PCR for the indicated transcripts. (J) Tumors were lysed in RIPA buffer, and lysates were subjected to Western blot. (K) Tumors were fixed, sectioned, and stained with H&E. The bar graphs showed the data of three independent experiments, presented as mean ± SEM. UD is undetectable. Statistics: unpaired two-tailed student's *t*-test or two-way ANOVA with multiple comparisons (∗∗*P* < 0.01, ∗∗∗*P* < 0.001).

To resolve this ambiguity, we first established tumors and then administered the virus (Fig. 6D). Raji cells were engrafted subcutaneously into the inguinal regions of 6-week-old female BALB/c Nude mice; five days later, when palpable tumors had formed, VRPs or PBS were injected intratumorally. Body weights remained indistinguishable across untreated, PBS-treated, and VRP-treated cohorts (Fig. 6E), yet VRP-infected tumors grew significantly more slowly (Fig. 6F–H).

To confirm that these effects were associated with the VRP infection and the decreased EBNA1 expression, we examined the viral infection products and the EBNA1 protein levels. Quantitative real-time PCR confirmed robust VRP replication, as both genomic and sub-genomic viral RNAs were readily detected (Fig. 6I). Western blot showed a pronounced drop in EBNA1 protein in VRP-treated tumors (Fig. 6J), and this loss coincided with marked up-regulation of Zta and gp350 transcripts (Fig. 6I), confirming a latent-to-lytic switch. Histological analysis (H&E) and WB revealed widespread cell death within VRP-infected tumors (Fig. 6K) and enhanced apoptotic cell death, respectively (Supporting Information Fig. S6A and S6B), consistent with entry into the EBV lytic cycle.

### 3.7 PLpro inhibits EBV tumor proliferation

Having established that SARS-CoV-2 PLpro degrades EBNA1 and reactivates EBV, we next asked whether PLpro alone could suppress EBV-driven tumor growth. Next, we interrogated PLpro for its inhibitory effect on EBV tumor proliferation. To achieve the expression of PLpro in EBV tumors, we constructed AAV9 encoding PLpro (AAV9-PL) and employed the same intratumor injection strategy used in the VRP infection assay (Fig. 7A).

**Figure 7:**
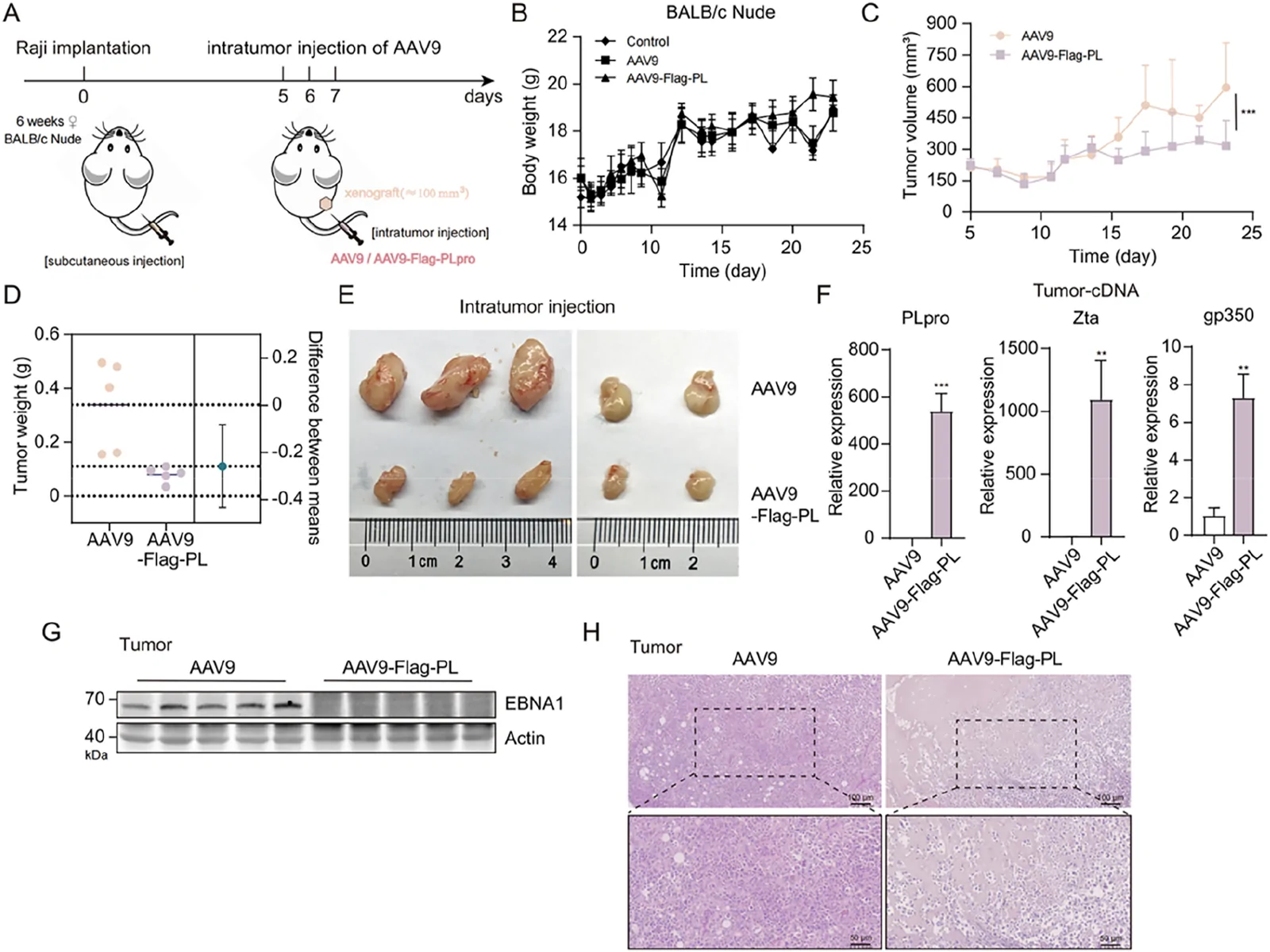
PLpro inhibits EBV tumor proliferation. (A) The schematic illustration of the Raji xenograft mouse model with the infection of AAV9 encoding Flag-PLpro (AAV9-Flag-PLpro), which is similar to the schematic illustration of Fig. 6D, with the exception of using AAV9 instead of VRPs for infection (*n* = 5). The body weight (B) and the tumor volume (C) were recorded for 23 days. At the endpoint of the experiment, tumors were collected the tumor weight was recorded (D and E), the PLpro, Zta and gp350 transcripts were quantified using quantitative real-time PCR (F), the expression of EBNA1 was analyzed by Western blot (G), and the cell death was assessed in H&E-stained tumor sections (H). The bar graphs showed the data of three independent experiments, presented as mean ± SEM. Statistics: unpaired two-tailed student's *t*-test or two-way ANOVA with multiple comparisons (∗∗*P* < 0.01, ∗∗∗*P* < 0.001).

Body weights remained indistinguishable among untreated, AAV9-control, and AAV9-PL mice (Fig. 7B), yet tumors receiving AAV9-PL were markedly smaller (Fig. 7C–E). Robust PLpro expression in treated tumors (Fig. 7F) correlated with a sharp drop in EBNA1 protein (Fig. 7G) and a concomitant rise in Zta and gp350 transcripts (Fig. 7F), confirming a latent-to-lytic switch. Extensive tumor-cell death (Fig. 7H, Fig. S6C and S6D) further validated productive lytic reactivation. Collectively, these data establish PLpro as the effector by which SARS-CoV-2 curtails EBV-associated tumor growth.

## 4 Discussion and conclusions

Our understanding of the impacts of SARS-CoV-2 on the infection and circulation of other viruses remains limited. It is reported that the incidence of newly diagnosed NPC cases in Singapore declined significantly in 2020^36^, indicating a possible impact of SARS-CoV-2 on EBV infection. Furthermore, SARS-CoV-2 may also affect the infection pattern of rhinovirus/enterovirus^37^^,^^38^. Despite growing evidence of these interactions, the molecular mechanisms underlying the impact of SARS-CoV-2 on other viruses remain poorly understood.

Our study revealed the interaction between SARS-CoV-2 and EBV. Besides EBV, many viruses were likely affected by SARS-CoV-2. For example, during the COVID-19 pandemic, influenza B/Yamagata probably became extinct^39^. As the largest known RNA virus, coronaviruses are highly versatile and encode over 30 viral proteins, many of whose functions are still not fully understood. Moreover, coronaviruses share similar infection routes with many viruses, suggesting their potential for extensive interaction with a wide range of viral pathogens.

Sixty-one years ago, EBV was discovered as the first human oncogenic virus. However, to date, there are no approved therapeutic vaccines or specific medicines targeting this virus^40^^,^^41^. In this study, we reported that SARS-CoV-2 infection can disrupt the EBV latent state through PLpro-mediated degradation of EBNA1. This disruption, caused by either SARS-CoV-2 infection or PLpro ectopic expression, can reactivate EBV infection and inhibit the proliferation of EBV-associated tumors. These results are supported by the reports about elevated EBV reactivation in COVID-19 patients^42^. Thus, our findings support the notion that targeting EBNA1 could be a potential strategy for anti-EBV therapy.

We acknowledge that the observed decrease in tumor cell proliferation may not be exclusively attributed to nsp3-mediated mechanisms. Other SARS-CoV-2 proteins, particularly 3CLpro, could potentially contribute to this phenotype. 3CLpro has been reported to cleave multiple host proteins involved in cell cycle regulation and apoptosis, including NLRP1, and its overexpression alone can induce cytotoxic effects in certain cell types^43^. Additionally, general viral-induced cytotoxicity resulting from cellular stress responses, ER stress, or innate immune activation cannot be ruled out as contributing factors^44^, ^45^, ^46^, ^47^.

While lytic reactivation represents a potential therapeutic strategy in EBV-driven malignancies, where inducing viral cytotoxicity may suppress tumor growth, this approach is not applicable to non-cancer settings. In immunocompromised or otherwise vulnerable populations, EBV reactivation poses significant clinical risks, including severe inflammatory responses and life-threatening lymphoproliferative disorders^48^.

This study has several limitations. First, we employed a VRP system rather than authentic SARS-CoV-2 due to biosafety constraints. While VRPs recapitulate viral replication and protein expression, they lack the S protein and cannot produce infectious progeny, potentially missing aspects of full viral pathogenesis. Second, our *in vivo* experiments were conducted in immunodeficient BALB/c Nude mice, which do not fully model the human immune response to either SARS-CoV-2 or EBV. The interplay between PLpro-mediated EBNA1 degradation and host immunity remains unexplored. Third, we focused on established cell lines and did not validate our findings in primary patient-derived EBV-positive tumor samples or organoid models, which may better reflect tumor heterogeneity. Fourth, the long-term consequences of SARS-CoV-2-induced EBV reactivation, whether beneficial through oncolytic effects or detrimental through enhanced viral transmission, remain unknown. Fifth, the co-infection of EBV and SARS-CoV-2 remains to be fully validated; While EBV cell lines and EBV-susceptible cells may express ACE2 receptors, the absence of suitable animal models presents a significant obstacle to confirming this hypothesis. Finally, while we demonstrated PLpro's therapeutic potential *via* AAV9 delivery, the translational feasibility of this approach, including delivery efficiency, off-target effects, and optimal dosing regimens, requires extensive preclinical optimization.

## Author contributions

Liubing Du: Conceptualization, Methodology, Validation, Formal analysis, Investigation, Data Curation, Writing (Original Draft, Review & Editing), Visualization, Project administration, Funding acquisition. Mengxuan Wang: Validation, Formal analysis, Investigation, Data Curation, Writing (Original Draft, Review & Editing).

Xue Liu: Software, Resources, Data Curation, Writing (Original Draft, Review & Editing). Ziliang Peng: Validation, Writing (Original Draft, Review & Editing). Zhihui Zhang: Validation. Zhanjia Zhang: Validation. Zhijuan Liang: Validation. Qingrong Sun: Validation. Jiaxin Zhuang: Resources. Kongyang Ma: Funding acquisition. Yao-Qing Chen: Methodology. Panpan Hou: Methodology. Hua Zhang: Resources. Deyin Guo: Conceptualization, Methodology, Formal analysis, Writing (Original Draft, Review & Editing), Supervision, Project administration, Funding acquisition. Ji-An Pan: Conceptualization, Methodology, Formal analysis, Writing (Original Draft, Review & Editing), Supervision, Project administration, Funding acquisition. Xiaoxue Peng: Conceptualization, Methodology, Formal analysis, Writing (Original Draft, Review & Editing), Supervision, Project administration, Funding acquisition.

## Conflicts of interest

The authors declare that they have no conflicts of interest.

## Appendix A Supporting information

The following is the Supporting Information to this article.
