# Supplementary material for: SARS-CoV-2 inhibits EBV-dependent tumor proliferation through PLpro-mediated degradation of EBNA1
Source: Acta Pharm Sin B. 2026 Jul 30;16(11):7418–33. doi: 10.1016/j.apsb.2026.07.042 (PMC13729527; doi:10.1016/j.apsb.2026.07.042)
Supplement: Supplementary file 1 [file mmc1.docx]

**Supporting Information for**

**Original article**

**SARS-CoV-2 inhibits EBV-dependent tumor proliferation through PLpro-mediated degradation of EBNA1**

**Liubing Du^a,b^, Mengxuan Wang^a^, Xue Liu^a^, Ziliang Peng^a^, Zhihui Zhang^a^, Zhanjia Zhang^a^, Zhijuan Liang^a^, Qingrong Sun^a^, Jiaxin Zhuang^a^, Kongyang Ma^c^, Yao-Qing Chen^f^, Panpan Hou^e^, Hua Zhang^c^, Deyin Guo^b,*^, Ji-An Pan^a,c,*^, Xiaoxue Peng^a,c,d,*^**

^a^*The Molecular Cancer Research Center, School of Medicine, Shenzhen Campus of Sun Yat-sen University, Shenzhen 518107, China*

^b^*The Division of Basic Research, Guangzhou National Laboratory, Bio-island, Guangzhou 510320, China*

^c^*The Center for Infection and Immunity Study, School of Medicine, Shenzhen Campus of Sun Yat-sen University, Shenzhen 518107, China*

^d^*Shenzhen Key Laboratory for Systems Medicine in Inflammatory Diseases, School of Medicine, Shenzhen Campus of Sun Yat-sen University, Shenzhen 518107, China*

^e^*State Key Laboratory of Respiratory Disease, National Clinical Research Center for Respiratory Disease, Guangzhou Institute of Respiratory Health, The First Affiliated Hospital of Guangzhou Medical University, Guangzhou 510120, China*

^f^*School of Public Health (Shenzhen), Shenzhen Campus of Sun Yat-sen University, Shenzhen 518107, China*

Received 22 September 2025; received in revised form 2 April 2026; accepted 19 April 2026

*Corresponding author.

E-mail addresses: [pengxx9@mail.sysu.edu.cn](mailto:pengxx9@mail.sysu.edu.cn) (Xiaoxue Peng), [panjan@mail.sysu.edu.cn](mailto:panjan@mail.sysu.edu.cn) (Ji-An Pan), [guodeyin@mail.sysu.edu.cn](mailto:guodeyin@mail.sysu.edu.cn) (Deyin Guo).

1. **Supporting figure**

**Figure S1** (A) The schematic of the PLpro activity reporter system. A fusion protein of polyubiquitin (UB) and firefly luciferase (Luci) is degraded in the proteasome. PLpro recognizes and cleaves the cleavage site (CS) inserted between UB and Luci, leading to the survival of firefly luciferase. The activity of firefly luciferase reflects the PLpro activity. (B) HEK293T cells were transfected with the UB-Luci plasmid, the Renilla luciferase plasmid, and either the control vector or the PLpro plasmid. Forty-eight hours post-transfection, the cells were collected and subjected to a dual-luciferase assay (*n* = 3). (C) HEK293T cells were transfected with the UB-2/3-Luci plasmid, the Renilla luciferase plasmid, and either the vector or the PLpro plasmid. Note that 2/3 indicates the cleavage site between nsp2 and nsp3. Forty-eight hours post-transfection, the cells were collected and subjected to a dual-luciferase assay (*n* = 3). (D) HEK293T cells were transfected with the UB-2/3-Luci plasmid, the Renilla luciferase plasmid, and either the WT PLpro plasmid or the PLpro plasmids with indicated mutations. Forty-eight hours post-transfection, the cells were collected and subjected to a dual-luciferase assay (*n* = 3). (E) HEK293T cells were transfected with the UB-CS-Luci plasmid, the Renilla luciferase plasmid, and the PLpro plasmids. Twenty‑four hours post-transfection, the cells were treated with GRL-0617 at 50 or 100 µmol/L for 48 h (*n* = 3). (F) The potential cleavage sites of the top 4 viruses in Fig. 1B were examined using the PLpro activity reporter system (*n* = 3). (G) HEK293T cells were transfected with the Flag-PL plasmid and the GFP-CAT or GFP-CS-CAT plasmid, which contains CS between nsp2 and nsp3 of SARS-CoV-2. Forty-eight hours post-transfection, cells were lysed, and cell lysates were subjected to Western blot analysis. (H) HEK293T cells were transfected with the GFP-CS-CAT plasmid and either the WT PLpro plasmid or various PLpro plasmids with indicated mutations. Forty-eight hours post-transfection, cells were lysed, and cell lysates were subjected to Western blot analysis. The bar graphs showed the data of three independent experiments, presented as mean ± SEM. Statistics: unpaired two-tailed student’s *t*-test or two-way ANOVA with multiple comparisons (**P* < 0.05, ***P* < 0.01, ****P* < 0.001).

**Figure S2** The decreased EBNA1 levels in Fig. 2F (A) and Fig. 2L (B) were calculated by subtracting the EBNA1 levels in samples containing PLpro, various PLpro mutants, or PLpro plus GRL-0617 from the control EBNA1 levels. Note: if the value was lower than zero, it was recorded as zero. Subsequently, the decreased EBNA1 levels were normalized to the corresponding levels of PLpro or PLpro mutants. These results reflect the influence of the expression level of PLpro on the inhibitory effect of PLpro mutations and GRL-0617. (C) HEK293T cells were transfected with the PL-HA plasmid and either Flag-EBNA1 or Flag-EBNA1 (CS-Mut) plasmids, then lysed and subjected to Western blot analysis. (D) Band intensities were quantified using Image Studio software on the Odyssey CLx system (*n* = 3). (E) HeLa cells transfected with GFP or GFP-nsp3 alone showed no Flag staining signal, confirming the specificity of the Flag antibody. (F) Similarly, HeLa cells transfected with Flag-EBNA1 alone showed no GFP signal. HEK293T cells were transfected with GFP-PL deletion mutants lacking the UBL, THUMB, FINGER, or PALM domain. Cytoplasmic and nuclear fractions were analyzed by Western blot (G), and band intensities were quantified using Image Studio software (Odyssey CLx) to calculate the subcellular distribution of each mutant (*n* = 3) (H). (I) ACE2 expression in Raji, C666-1, and SNU-719 cells was examined by Western blot, using HEK293T cells overexpressing ACE2 as a control. The bar graphs showed the data of three independent experiments, presented as mean ± SEM. Statistics: unpaired two-tailed student’s *t*-test or two-way ANOVA with multiple comparisons (***P* < 0.01, ****P* < 0.001).

**Figure S3** (A) The packaging strategy of viral replicon particles (VRPs). VSV-G plasmid and SARS-CoV-2 replicon lacking the S gene were transfected into Huh7.5 cells using polyethyleneimine (PEI). 48–96 h post-transfection, the supernatant was harvested and stored at –80 °C for subsequent use. Raji (B), C666-1 (C), and SNU-719 (D) cells were infected with VRPs. At the indicated days post-infection, cells were collected, total RNA was extracted, and the indicated genes were analyzed by quantitative real-time PCR (*n* = 3). EBV copy numbers in C666-1 (E) and SNU-719 (F) cells were determined by quantitative real-time PCR (*n* = 3). Data are presented from at least three independent experiments with consistent results. The bars are represented as mean ± SEM. Statistics: unpaired two-tailed student’s *t*-test or two-way ANOVA with multiple comparisons (**P* < 0.05, ***P* < 0.01, ****P* < 0.001).

**Figure S4** (A) Raji cells with or without EBNA1 overexpression were infected with VRPs. Cells were collected, total RNA was extracted, and the indicated genes were analyzed by quantitative real-time PCR (qRT-PCR) (*n* = 3). (B) Total RNA was extracted from Raji cells with or without 3CLpro overexpression, and the indicated genes were analyzed by qRT-PCR (*n* = 3). (C) Total RNA was extracted from Raji cells expressing shNTC or shIRF3, and the indicated genes were subsequently analyzed by qRT-PCR (*n* = 3). Data are presented from at least three independent experiments with consistent results. The bars are represented as mean ± SEM. Statistics: unpaired two-tailed student’s *t*-test (**P* < 0.05, ***P* < 0.01, ****P* < 0.001).

**Figure S5** Raji cells were either left untreated or infected with VRPs and observed under a microscope (A). 1×10^7^ cells untreated or infected with VRPs were subcutaneously injected into the inguinal regions of the 6-week-old female mice (B). The body weight (C) and the tumor volume (D) were recorded for 22 days. At the endpoint of the experiment, tumors were collected (E) and photographed (F). Statistics: two-way ANOVA with multiple comparisons (****P* < 0.001).

**Figure S6** Tumors in Fig. 6 (A and B) or Fig. 7 (C and D) were subjected to Western blot assay. The expression levels of PARP, cleaved PARP, caspase-7, and cleaved caspase-7 were analyzed. The intensity of bands was quantified using Image Studio of Odyssey CLx, and the cleavage efficiency was calculated (*n* = 5) (C and D). Statistics: unpaired two-tailed student’s *t*-test or two-way ANOVA with multiple comparisons (**P* < 0.05, ****P* < 0.001).

**2. Supporting table**

**Table S1** The sequences of primers used in this study.

| Name | Sequence (5′–3′) |
| --- | --- |
| Primers for the construction of LPC-related plasmids | |
| Homo-*nsp3* f | TATGGGTACTCGAGCCCGCCCTTCAGGGC |
| Homo-*nsp3* r | CCAAGCTGGCTAGCATGGCCCCCACCAAG |
| *Hind3*-PL f | CCGAAGCTTACCATGCGTGAGGTTCGTACCATCAAGG |
| *Xho1*-PL r | CCCTCGAGCTTGTAGGTCACCGGTTTAATGGTG |
| Primers for mutagenesis | |
| PLC111S f | CAACAACTCCTATCTGGCGACCGCGCTG |
| PLC111S r | CCAGATAGGAGTTGTTGTCCGCCCATTTGATG |
| PLH272A f | GTGCGGTGCCTATAAACACATCACCAGCAAGGAG |
| PLH272A r | GTGTTTATAGGCACCGCACTGATAGTTGCCGG |
| PLD286A f | CTGCATTGCTGGTGCGCTGCTGACC |
| PLD286A r | GCACCAGCAATGCAGTACAGGGTCTCCTTGC |
| Bgl2-PL1 f | GAAGATCTGAGGTTCGTACCATCAAGGTGT |
| PL1-Xho1 r | CCCTCGAGTTAGGTGGTGTGATAGTATTCGAACGC |
| Bgl2-PL2 f | GAAGATCTGACCCGAGCTTTCTGGGTCG |
| PL2-Xho1 r | CCCTCGAGTTACAGGTTCGCGTGTTGGAACA |
| Bgl2-PL3 f | GAAGATCTGACAGCTGCAAGCGTGTTCT |
| PL3-Xho1 r | CCCTCGAGTTATTCCTGTTGCACCAGATATTTGG |
| Bgl2-PL4 f | GAAGATCTAGCCCGTTCGTTATGATGAGC |
| PL4-Xho1 r | CCCTCGAGTTATTTAATGGTGGTGGTGTAGCTGTTC |
| CS-Mut f1 | GGAGCCGCAAGACCAGGAGCCCCGG |
| CS-Mut r1 | GTCTTGCGGCTCCTCGTCCTCGTCCTCTTCC |
| CS-Mut f2 | CCGCTGTGTTCGTATATGGAGCTAGTAAGACCTCCCTTTACAACC |
| CS-Mut r2 | TTAGGTTGTAAAGGGAGGTCTTACTAGCTCCATATACGAACACA |
| ▲PL1 f | CAGATCTGACCCGAGCTTTCTGGGTC |
| ▲PL1 r | TCGGGTCAGATCTGAGTCCGGACTTGTACAG |
| ▲PL2 f | CACCACCGACAGCTGCAAGCGTGTTC |
| ▲PL2 r | AGCTGTCGGTGGTGTGATAGTATTCGAACGC |
| ▲PL3 f | GAACCTGAGCCCGTTCGTTATGATGAGC |
| ▲PL3 r | ACGGGCTCAGGTTCGCGTGTTGGAACA |
| ▲PL4 f | ACAGGAATAACTCGAGCATGCATCTAGAGGG |
| ▲PL4 r | CGAGTTATTCCTGTTGCACCAGATATTTGGTC |
| 3CL f | CGACGCGTGCCACCATGAGCGGTTTCCGTAAGATGGC |
| 3CL r | ACGCGTCGACTTACTTGTACAGCTCGTCCATGC |
| *EBNA1* f | CAAGGGAGGATCCATGTCTGACGAGGGTCCAGG |
| *EBNA1* r | TCGACTCACTCCTGACCTTCCTCACCCT |
| Primers for cloning pLKO.1-shRNA | |
| shEBNA1 f | CCGGAACGGAGATGACGGAGATGAACTCGAGTTCATCTCCGTCATCTCCGTTTTTTTG |
| shEBNA1 r | AATTCAAAAAAACGGAGATGACGGAGATGAACTCGAGTTCATCTCCGTCATCTCCGTT |
| shEBNA1Seq f | CGGAGATGACGGAGATGAA |
| U6 f | GAGGGCCTATTTCCCATGAT |
| cPPT r | TATGTCTACTATTCTTTCCCCTGC |
| shIRF3 f | CCGGAAGCAGGAGGATTTCGGAATCTTCTCGAGAAGATTCCGAAATCCTCCTGCTTTTTTTG |
| shIRF3 r | AATTCAAAAAAAGCAGGAGGATTTCGGAATCTTCTCGAGAAGATTCCGAAATCCTCCTGCTT |
| Primers for cloning cleavage sites of PLpro | |
| *Hind3*-CS f | AGCTTTTCACACTCAAAGGCGGTGCACCAACAAAGGTTA |
| *Hind3*-CS r | AGCTTAACCTTTGTTGGTGCACCGCCTTTGAGTGTGAAA |
| *Hind3*-CS1 f | AGCTTGCAGGAGCAGGAGGAGGGGCAGGAGCAGGAGGAA |
| *Hind3*-CS1 r | AGCTTTCCTCCTGCTCCTGCCCCTCCTCCTGCTCCTGCA |
| *Hind3*-CS2 f | AGCTTGCAGGAGCAGGAGGAGGGGCAGGAGGGGCAGGAA |
| *Hind3*-CS2 r | AGCTTTCCTGCCCCTCCTGCCCCTCCTCCTGCTCCTGCA |
| *Hind3*-CS3 f | AGCTTAGGCGCAAAAAAGGAGGGTGGTTTGGAAAGCATA |
| *Hind3*-CS3 r | AGCTTATGCTTTCCAAACCACCCTCCTTTTTTGCGCCTA |
| *Hind3*-CS4 f | AGCTTCGAGGACGAGGAGGCGGAAGACCAGGAGCCCCGA |
| *Hind3*-CS4 r | AGCTTCGGGGCTCCTGGTCTTCCGCCTCCTCGTCCTCGA |
| *Hind3*-CS5 f | AGCTTCCAGGAGCCCCGGGCGGCTCAGGATCAGGGCCAA |
| *Hind3*-CS5 r | AGCTTTGGCCCTGATCCTGAGCCGCCCGGGGCTCCTGGA |
| *Hind3*-CS6 f | AGCTTCCAGAAGGCTCCGGCGGCAGTGGACCTCAAAGAA |
| *Hind3*-CS6 r | AGCTTTCTTTGAGGTCCACTGCCGCCGGAGCCTTCTGGA |
| *Hind3*-CS7 f | AGCTTCCTCAAAGAAGAGGGGGTGATAACCATGGACGAA |
| *Hind3*-CS7 r | AGCTTTCGTCCATGGTTATCACCCCCTCTTCTTTGAGGA |
| *Hind3*-CS8 f | AGCTTAAAGGGACCCACGGTGGAACAGGAGCAGGAGCAA |
| *Hind3*-CS8 r | AGCTTTGCTCCTGCTCCTGTTCCACCGTGGGTCCCTTTA |
| *Hind3*-CS9 f | AGCTTGCAGGAGCAGGAGGTGGAGGCCGGGGTCGAGGAA |
| *Hind3*-CS9 r | AGCTTTCCTCGACCCCGGCCTCCACCTCCTGCTCCTGCA |
| *Hind3*-CS10 f | AGCTTGGCCGGGGTCGAGGAGGCAGTGGAGGCCGGGGTA |
| *Hind3*-CS10 r | AGCTTACCCCGGCCTCCACTGCCTCCTCGACCCCGGCCA |
| *Hind3*-CS11 f | AGCTTGAAAGAGCCAGGGGGGGAAGTCGTGAAAGAGCCA |
| *Hind3*-CS11 r | AGCTTGGCTCTTTCACGACTTCCCCCCCTGGCTCTTTCA |
| *Hind3*-CS12 f | AGCTTTACCACCAAGAAGGTGGCCCAGATGGTGAGCCTA |
| *Hind3*-CS12 r | AGCTTAGGCTCACCATCTGGGCCACCTTCTTGGTGGTAA |
| *Hind3*-CS13 f | AGCTTGGTCAGGGTGATGGAGGCAGGCGCAAAAAAGGAA |
| *Hind3*-CS13 r | AGCTTTCCTTTTTTGCGCCTGCCTCCATCACCCTGACCA |
| *Hind3*-CS14 f | AGCTTCATCGTGGTCAAGGAGGTTCCAACCCGAAATTTA |
| *Hind3*-CS14 r | AGCTTAAATTTCGGGTTGGAACCTCCTTGACCACGATGA |
| *Hind3*-CS15 f | AGCTTGTGTTCGTATATGGAGGTAGTAAGACCTCCCTTA |
| *Hind3*-CS15 r | AGCTTAAGGGAGGTCTTACTACCTCCATATACGAACACA |
| *BamH1*-CS f | GATCCTTCACACTCAAAGGCGGTGCACCAACAAAGGTTG |
| *BamH1*-CS r | GATCCAACCTTTGTTGGTGCACCGCCTTTGAGTGTGAAG |
| *BamH1*-CS1 f | GATCCGCAGGAGCAGGAGGAGGGGCAGGAGCAGGAGGAG |
| *BamH1*-CS1 r | GATCCTCCTCCTGCTCCTGCCCCTCCTCCTGCTCCTGCG |
| *BamH1*-CS4 f | GATCCCGAGGACGAGGAGGCGGAAGACCAGGAGCCCCGG |
| *BamH1*-CS4 r | GATCCCGGGGCTCCTGGTCTTCCGCCTCCTCGTCCTCGG |
| *BamH1*-CS5 f | GATCCCCAGGAGCCCCGGGCGGCTCAGGATCAGGGCCAG |
| *BamH1*-CS5 r | GATCCTGGCCCTGATCCTGAGCCGCCCGGGGCTCCTGGG |
| *BamH1*-CS9 f | GATCCGCAGGAGCAGGAGGTGGAGGCCGGGGTCGAGGAG |
| *BamH1*-CS9 r | GATCCTCCTCGACCCCGGCCTCCACCTCCTGCTCCTGCG |
| *BamH1*-CS11 f | GATCCGAAAGAGCCAGGGGGGGAAGTCGTGAAAGAGCCG |
| *BamH1*-CS11 r | GATCCGGCTCTTTCACGACTTCCCCCCCTGGCTCTTTCG |
| *BamH1*-CS15 f | GATCCGTGTTCGTATATGGAGGTAGTAAGACCTCCCTTG |
| *BamH1*-CS15 r | GATCCAAGGGAGGTCTTACTACCTCCATATACGAACACG |
| EBna1-CS1 f | AGCTTCGAGGACGAGGAGGCGGA AGACCAGGAGCCCCGA |
| EBna1-CS1 r | AGCTTCGGGGCTCCTGGTCTTCC GCCTCCTCGTCCTCGA |
| EBna1-CS2 f | AGCTTGTGTTCGTATATGGAGGTA GTAAGACCTCCCTTA |
| EBna1-CS2 r | AGCTTAAGGGAGGTCTTACTACCTCCATATACGAACACA |
| *BORF1*-CS1 f | AGCTTGTGGCGGTCGCCGGGGGTGGCAGTGGAGGGGGGA |
| *BORF1*-CS1 r | AGCTTCCCCCCTCCACTGCCACCCCCGGCGACCGCCACA |
| *BORF1*-CS2 f | AGCTTGCGTGGCTCTCCGGCGGCGCCGGGGGTGGCGGTA |
| *BORF1*-CS2 r | AGCTTACCGCCACCCCCGGCGCCGCCGGAGAGCCACGCA |
| *BHLF1*-CS f | AGCTTGGGGCACGCCCTGGGGGAGGGAACCGGGTGGGAA |
| *BHLF1*-CS r | AGCTTTCCCACCCGGTTCCCTCCCCCAGGGCGTGCCCCA |
| *BBLF3*-CS1 f | AGCTTCTCAGGCGCAGCGGGGGGCTCATTGCTATGCTCA |
| *BBLF3*-CS1 r | AGCTTGAGCATAGCAATGAGCCCCCCGCTGCGCCTGAGA |
| *BBLF3*-CS2 f | AGCTTTGCACGCGGGAAGGCGGCGAGGGAGGGGGGGAGA |
| *BBLF3*-CS2 r | AGCTTCTCCCCCCCTCCCTCGCCGCCTTCCCGCGTGCAA |
| *BBLF3*-CS3 f | AGCTTCGGGATGTGATGGGTGGAGAGGCCACCGTGGCCA |
| *BBLF3*-CS3 r | AGCTTGGCCACGGTGGCCTCTCCACCCATCACATCCCGA |
| *BPLF1*-CS1/2 f | AGCTTGTGGTCCTGAGGGGCGGTGGGCGCGTGCACATAA |
| *BPLF1*-CS1/2 r | AGCTTTATGTGCACGCGCCCACCGCCCCTCAGGACCACA |
| *BPLF1*-CS3 f | AGCTTGACCTGCTCTCGGGCGGAGATCAGGAGGCGGGGA |
| *BPLF1*-CS3 r | AGCTTCCCCGCCTCCTGATCTCCGCCCGAGAGCAGGTCA |
| *BPLF1*-CS4 f | AGCTTCTGGCCTTAGCTGGGGGCGCGGGTGCCTGGCTGA |
| *BPLF1*-CS4 r | AGCTTCAGCCAGGCACCCGCGCCCCCAGCTAAGGCCAGA |
| *BPLF1*-CS5 f | AGCTTAAGCGGGTCGCGGGGGGCAAGGAGACCGTCGCAA |
| *BPLF1*-CS5 r | AGCTTTGCGACGGTCTCCTTGCCCCCCGCGACCCGCTTA |
| *BPLF1*-CS6 f | AGCTTCTAGCGAGAGATGGGGGTTTGGGAGACCTCCTGA |
| *BPLF1*-CS6 r | AGCTTCAGGAGGTCTCCCAAACCCCCATCTCTCGCTAGA |
| *BPLF1*-CS7 f | AGCTTCAGCAGTTGGGAGGAGGGGAGACGGACCAGGGGA |
| *BPLF1*-CS7 r | AGCTTCCCCTGGTCCGTCTCCCCTCCTCCCAACTGCTGA |
| *BPLF1*-CS8 f | AGCTTGAGGTGGTGACAGGAGGGTCGACCTCCGGCGACA |
| *BPLF1*-CS8 r | AGCTTGTCGCCGGAGGTCGACCCTCCTGTCACCACCTCA |
| UL44-CS1 f | AGCTTAAATCGCGTGGCGGCGGCGGTGGTAGCGGCAGCA |
| UL44-CS1 r | AGCTTGCTGCCGCTACCACCGCCGCCGCCACGCGATTTA |
| UL44-CS2 f | AGCTTGGCGGTCTGGGAGGAGGTGGCGGCGGTGGCGGCA |
| UL44-CS2 r | AGCTTGCCGCCACCGCCGCCACCTCCTCCCAGACCGCCA |
| UL44-CS3 f | AGCTTCACGACCGCGGTGGCGGCGGTGGTTCCGGTACGA |
| UL44-CS3 r | AGCTTCGTACCGGAACCACCGCCGCCACCGCGGTCGTGA |
| UL112-CS1 f | AGCTTCTCTTTGTACGCGGCGGTA TCGTCTTTAACAAAA |
| UL112-CS1 r | AGCTTTTTGTTAAAGACGATACCG CCGCGTACAAAGAGA |
| UL112-CS2 f | AGCTTAATACCCGAGGAGGCGGCGGCGGTGGTGGCGGTA |
| UL112-CS2 r | AGCTTACCGCCACCACCGCCGCCGCCTCCTCGGGTATTA |
| UL57-CS1 f | AGCTTGTGCGACTGCACGGCGGCGAGGCGGTGCGCGTGA |
| UL57-CS1 r | AGCTTCACGCGCACCGCCTCGCCGCCGTGCAGTCGCACA |
| UL57-CS2 f | AGCTTGACGGGGTTCCGGGCGGCGGTGCTGGCGGGGGTA |
| UL57-CS2 r | AGCTTACCCCCGCCAGCACCGCCGCCCGGAACCCCGTCA |
| UL57-CS3 f | AGCTTCGGGACGTGAGCGGGGGCCCGAGCGACGGTCTGA |
| UL57-CS3 r | AGCTTCAGACCGTCGCTCGGGCCCCCGCTCACGTCCCGA |
| UL57-CS4/5 f | AGCTTGACGGTCTGGGCGGCGGTCGTGGTGGTGGGGGTA |
| UL57-CS4/5 r | AGCTTACCCCCACCACCACGACCGCCGCCCAGACCGTCA |
| UL57-CS6 f | AGCTTTCCGGCGTCGGGGGTGGCGGTCTTCTTCTCGGCA |
| UL57-CS6 r | AGCTTGCCGAGAAGAAGACCGCCACCCCCGACGCCGGAA |
| UL51-CS1 f | AGCTTGGTGGGCTCGAGGGGGGAGGTGGTGACGACGAGA |
| UL51-CS1 r | AGCTTCTCGTCGTCACCACCTCCCCCCTCGAGCCCACCA |
| UL146-CS1 f | AGCTTTTGTATCTAGGCGGAGGAA AAAAACATGGACCTA |
| UL146-CS1 r | AGCTTAGGTCCATGTTTTTTTCCT CCGCCTAGATACAAA |
| UL76-CS1 f | AGCTTAACCGCCTGTTGGGAGGC CTTTTCCCACCCGAGA |
| UL76-CS1 r | AGCTTCTCGGGTGGGAAAAGGCCTCCCAACAGGCGGTTA |
| UL76-CS2 f | AGCTTCTGCCGCGCGGTGGCGGTCAAGTCTGGAGCGTAA |
| UL76-CS2 r | AGCTTTACGCTCCAGACTTGACCGCCACCGCGCGGCAGA |
| UL76-CS3/4 f | AGCTTTCGGCGGTTGCTGGAGGACGCGGCGGTGGCAATA |
| UL76-CS3/4 r | AGCTTATTGCCACCGCCGCGTCCTCCAGCAACCGCCGAA |
| UL76-CS5 f | AGCTTCGTGTCCGAGGCGGAGGCGCTGTTGAACCAGCAA |
| UL76-CS5 r | AGCTTTGCTGGTTCAACAGCGCCTCCGCCTCGGACACGA |
| UL80-CS1 f | AGCTTCAGCACCGTCACGGGGGCAGCGGCGGACACAACA |
| UL80-CS1 r | AGCTTGTTGTGTCCGCCGCTGCCCCCGTGACGGTGCTGA |
| UL80-CS2 f | AGCTTGCACTTCTATCCGGAGGTGCCAAGGTAGCTGAGA |
| UL80-CS2 r | AGCTTCTCAGCTACCTTGGCACCTCCGGATAGAAGTGCA |
| UL102-CS1 f | AGCTTTTGGATCTGGGTGGCGGCCGTGCCGAGCCGCGGA |
| UL102-CS1 r | AGCTTCCGCGGCTCGGCACGGCCGCCACCCAGATCCAAA |
| UL102-CS2 f | AGCTTCGCAAACTTTTTGGCGGCGACGATCCGGGTCCCA |
| UL102-CS2 r | AGCTTGGGACCCGGATCGTCGCCGCCAAAAAGTTTGCGA |
| RL2-CS1 f | AGCTTCAGGGCGTGGATGGGGGGGCGGTCTCGGGGGGGA |
| RL2-CS1 r | AGCTTCCCCCCCGAGACCGCCCCCCCATCCACGCCCTGA |
| RL2-CS2 f | AGCTTGGGGCGGTCTCGGGGGGGAGCCCCCCCCGCGAGA |
| RL2-CS2 r | AGCTTCTCGCGGGGGGGGCTCCCCCCCGAGACCGCCCCA |
| RL2-CS3 f | AGCTTCCCCCCGTGACGGGCGGGGCGTCTCACGCAGCCA |
| RL2-CS3 r | AGCTTGGCTGCGTGAGACGCCCCGCCCGTCACGGGGGGA |
| RL2-CS4 f | AGCTTCCGGGCGTGGAGGGTGGGCACGGGCCCTCCCGCA |
| RL2-CS4 r | AGCTTGCGGGAGGGCCCGTGCCCACCCTCCACGCCCGGA |
| US5-CS f | AGCTTCCCCTCGTAGTCGGTGGGCTGTGCGCCGTAGTCA |
| US5-CS r | AGCTTGACTACGGCGCACAGCCCACCGACTACGAGGGGA |
| UL36-CS1 f | AGCTTTGTTTGGTCGAGGGGGGCGAGTGGACGCGCGCGA |
| UL36-CS1 r | AGCTTCGCGCGCGTCCACTCGCCCCCCTCGACCAAACAA |
| UL36-CS2 f | AGCTTGAGGGCAGGTTCGGGGGCCTGTTGCACGCCGAAA |
| UL36-CS2 r | AGCTTTTCGGCGTGCAACAGGCCCCCGAACCTGCCCTCA |
| UL36-CS3 f | AGCTTCTGCGGCTGGCGGGGGGGCTGCTGGAGCGGGCCA |
| UL36-CS3 r | AGCTTGGCCCGCTCCAGCAGCCCCCCCGCCAGCCGCAGA |
| UL36-CS4 f | AGCTTGGCCGCGTTCGCGGGGGGGCCTGGAAATCGCCCA |
| UL36-CS4 r | AGCTTGGGCGATTTCCAGGCCCCCCCGCGAACGCGGCCA |
| UL36-CS5 f | AGCTTCTGCGAGTGAGCGGAGGCCCGGGGCCCCTGGTGA |
| UL36-CS5 r | AGCTTCACCAGGGGCCCCGGGCCTCCGCTCACTCGCAGA |
| UL36-CS6 f | AGCTTCCGTTTGTGGCGGGCGGCGAGGACGTCCTCGCCA |
| UL36-CS6 r | AGCTTGGCGAGGACGTCCTCGCCGCCCGCCACAAACGGA |
| UL36-CS7 f | AGCTTACGGTTTTGCCCGGGGGCGAAAACCGCCGCCCCA |
| UL36-CS7 r | AGCTTGGGGCGGCGGTTTTCGCC CCCGGGCAAAACCGTA |
| UL36-CS8 f | AGCTTTTGACGCTGGAGGGCGGTGTTGCGCCCGGAGGCA |
| UL36-CS8 r | AGCTTGCCTCCGGGCGCAACACCGCCCTCCAGCGTCAAA |
| RL2-CS f | AGCTTGCGCCCCGGCGCGGGGGCGGCGGTGCGGGGGCGA |
| RL2-CS r | AGCTTCGCCCCCGCACCGCCGCCCCCGCGCCGGGGCGCA |
| UL36-CS1 f | AGCTTTGTCTGGTGGAGGGGGGCGAGTGGACGCGCGCGA |
| UL36-CS1 r | AGCTTCGCGCGCGTCCACTCGCCCCCCTCCACCAGACAA |
| UL36-CS2 f | AGCTTCCGTACGTCGACGGGGGCGGGCTGTCCCCCGAGA |
| UL36-CS2 r | AGCTTCTCGGGGGACAGCCCGCCCCCGTCGACGTACGGA |
| UL36-CS3 f | AGCTTGGCCGCGTCCGGGGGGGCGCCTGGAAGTCCCCCA |
| UL36-CS3 r | AGCTTGGGGGACTTCCAGGCGCCCCCCCGGACGCGGCCA |
| UL36-CS4 f | AGCTTTACGCCAGGGCCGGCGGCCAGACGGGCGCGGGCA |
| UL36-CS4 r | AGCTTGCCCGCGCCCGTCTGGCCGCCGGCCCTGGCGTAA |
| UL36-CS5 f | AGCTTTATCTGCGCGCCGGCGGAGACCCGGGGCCCA |
| UL36-CS5 r | AGCTTGGGCCCCGGGTCTCCGCCGGCGCGCAGATAA |
| UL36-CS6 f | AGCTTACGTTTTTGGACGGAGGCCCCCCGACCCGGGGCA |
| UL36-CS6 r | AGCTTGCCCCGGGTCGGGGGGCCTCCGTCCAAAAACGTA |
| UL36-CS7 f | AGCTTGACTCGCGCCCCGGCGGGCCGGACGCCGCCCTGA |
| UL36-CS7 r | AGCTTCAGGGCGGCGTCCGGCCCGCCGGGGCGCGAGTCA |
| UL36-CS8 f | AGCTTCGGGACCTTGCGGGGGGGGATGGCGGATCGGGTA |
| UL36-CS8 r | AGCTTACCCGATCCGCCATCCCCCCCCGCAAGGTCCCGA |
| UL36-CS9 f | AGCTTGCGGTCGTCCCCGGGGGCGACCGCCGCCGCGGCA |
| UL36-CS9 r | AGCTTGCCGCGGCGGCGGTCGCCCCCGGGGACGACCGCA |
| UL36-CS10 f | AGCTTTGGGTCGTGCCCGGAGGCCCGGTTGCCCGCCGCA |
| UL36-CS10 r | AGCTTGCGGCGGGCAACCGGGCCTCCGGGCACGACCCAA |
| RS1-CS1 f | AGCTTCGCGTGCTGTACGGCGGGCTGGGCGACAGCCGCA |
| RS1-CS1 r | AGCTTGCGGCTGTCGCCCAGCCCGCCGTACAGCACGCGA |
| RS1-CS2 f | AGCTTCTGCGCGTGGCCGGCGGCAGCGAGGCCGCCGTGA |
| RS1-CS2 r | AGCTTCACGGCGGCCTCGCTGCCGCCGGCCACGCGCAGA |
| RS1-CS3 f | AGCTTGCGGGCCGCGCCGGGGGCGGGCCCCCCCCGGAGA |
| RS1-CS3 r | AGCTTCTCCGGGGGGGGCCCGCCCCCGGCGCGGCCCGCA |
| RS1-CS4 f | AGCTTGACGCCGTGCGCGGCGGCCCGGCGGAGCTGCGCA |
| RS1-CS4 r | AGCTTGCGCAGCTCCGCCGGGCCGCCGCGCACGGCGTCA |
| US5-CS f | AGCTTCTCATCCGTTCCGGGGGCTTTGCCGTCCCCCTCA |
| US5-CS r | AGCTTGAGGGGGACGGCAAAGCCCCCGGAACGGATGAGA |
| Primers for PCR/RT-PCR | |
| Leader f | CCTTCCCAGGTAACAAACCAACC |
| Leader r | CTCTCCATCTTACCTTTCGGTCAC |
| Npro r | CAGTATTATTGGGTAAACCTTGGGGC |
| Mpro r | GCATAGGCAAATTGTAGAAGACAAATCC |
| Zta f | CAGCCAGAATCGCTGGAGGAAT |
| Zta r | CTGCTTCAACAGGAGGCGCA |
| Rta f | CAGCCGGGAGTGTGGAGAT |
| Rta r | CGTAGCGCATCACTATAGGGCA |
| EBNA1 f | CGTGGTCAAGGAGGTTCCAA |
| EBNA1 r | TCCAAAGGGGAGACGACTCA |
| Flag-PL f | CATGACATCGATTACAAGGATGACG |
| Flag-PL r | CTCGTGGCTGTTGTGCGGTTTG |
| 3CL f | CCGAAGCTTACCATGAGCGGTTTCCGTAAGATGGC |
| 3CL r | CCCTCGAGTTGAAAGGTCACACCGCTGC |
| GFP f | TACGGCAAGCTGACCCTGAAG |
| GFP r | TGAAGCACTGCACGCCGTAGG |
| qHomo *GAPDH* f | GGAGCGAGATCCCTCCAAAAT |
| qHomo *GAPDH* r | GGCTGTTGTCATACTTCTCATGG |
| qMus *GAPDH* f | GACGTGCCGCCTGGAGA |
| qMus *GAPDH* r | GAAGAGTGGGAGTTGCTGTTGAA |
| qgp350 f | CCAAGACACTCATTATCACACGAACGG |
| qgp350 r | CTGTCGTTGTATTGGGATCAGCAAATC |
| IRF3 f | CTGATACCCAGGAAGACATTCTGG |
| IRF3 r | GAACTCCCACTCTTCCCCCG |

The optimized coding sequence of EBNA1

atgtctgacgagggtccaggtacaggacctggaaatggcctaggagagaagggagacacatctggaccagaaggctccggcggcagtggacctcaaagaagaggtggtgataaccatggacgaggacggggaagaggacgaggacgaggaggcggaagaccaggagccccgggtggctcaggatcaggtccaagacatagagatggtgtccggagacctcaaaaacgtccaagttgcattggctgcaaaggtacccacggtggaacaggcgcaggagcaggcgctggtggagcaggcgcaggaggtgcaggagcaggcggtggtgcaggcgcaggtggaggtgcaggtggagcaggcggtgcaggaggcgcaggcgcaggtggcggtgcaggcgcaggaggtggcgcaggaggtgcaggcggtgcaggagcaggcggaggcgcaggagcaggtggcggtgcaggaggcgcaggcgcaggaggtggtgcaggcggtgcaggtggtgcaggagcaggaggtggtgcaggcgcaggtggaggcgcaggcggtgcaggagcaggcggtggcgcaggaggtgcaggtggtgcaggagcaggtggcggtgcaggtgcaggcggtgcaggcggtgcaggcggtgcaggagcaggcggtgcaggagcaggtggcggtgcaggtggtgcaggtggagcaggagcaggaggtgcaggagcaggtggtgcaggagcaggtggtgcaggcgcaggaggagcaggtggagcaggagcaggaggtgcaggcggtgcaggagcaggcggtgcaggtggtgcaggagcaggtggaggtgcaggcggtgcaggagcaggcggtggagcaggaggtgcaggagcaggtggagcaggaggtgcaggagcaggtggtgcaggcggtgcaggagcaggtggtgcaggcggtgcaggagcaggtggcggtgcaggagcaggaggtgcaggagcaggaggtggaggccggggtcgaggaggcagtggaggccggggtcgaggaggtagtggaggccggggtcgaggaggtagtggaggccgccggggtagaggacgtgaaagagccaggggtggaagtcgtgaaagagccaggggtagaggtcgtggacgtggagaaaagaggcctaggagtcctagtagtcagtcatcatcatccggttctccaccgcgcaggcctcctccaggtagaaggccattcttccaccctgtaggtgaagccgattatttcgaataccaccaagaaggtggcccagatggtgagcctgacgtgcctccgggagcgatagagcagggccctgcagatgacccaggagaaggcccaagcactggacctcggggtcagggtgatggaggcaggcgcaaaaaaggaggttggtttggaaagcatcgtggtcaaggaggttccaacccgaaatttgagaacattgcagaaggtttaagagctctcctggctaggagtcacgtagaaaggactaccgacgaaggaacttgggtcgccggtgtgttcgtatatggaggtagtaagacctccctttacaacctaaggcgaggaactgcccttgctattccacaatgtcgtcttacaccattgagtcgtctcccttttggaatggcccctggacctggcccacaacctggcccgctaagggagtccattgtctgttatttcatggtctttttacaaactcatatatttgctgaggttttgaaggatgcgattaaggaccttgttatgacaaagcctgctcctacctgcaatatcagggtgactgtgtgcagctttgacgatggagtagatttgcctccttggtttccacctatggtggaaggtgctgccgcggagggtgatgacggagatgacggagatgaaggaggtgatggagatgagggtgaggaaggtcaggag
